# Phenotypic Heterogeneity Affects *Stenotrophomonas maltophilia* K279a Colony Morphotypes and β-Lactamase Expression

**DOI:** 10.3389/fmicb.2015.01373

**Published:** 2015-12-02

**Authors:** Ebrahim M. Abda, Dagmar Krysciak, Ines Krohn-Molt, Uwe Mamat, Christel Schmeisser, Konrad U. Förstner, Ulrich E. Schaible, Thomas A. Kohl, Stefan Nieman, Wolfgang R. Streit

**Affiliations:** ^1^Department of Microbiology and Biotechnology, Biocenter Klein Flottbek – University of HamburgHamburg, Germany; ^2^Priority Program Asthma and Allergy, Leibniz-Center for Medicine and Biosciences – Research Center BorstelBorstel, Germany; ^3^Core Unit Systems Medicine, University of WürzburgWürzburg, Germany; ^4^Priority Program Infections, Leibniz-Center for Medicine and Biosciences – Research Center BorstelBorstel, Germany; ^5^German Center for Infection ResearchBorstel, Germany

**Keywords:** phenotypic heterogeneity, *Stenotrophomonas maltophilia*, K279a, β-lactamases, colony morphotypes, antibiotic resistance, RNA-seq

## Abstract

Phenotypic heterogeneity at the cellular level in response to various stresses, e.g., antibiotic treatment has been reported for a number of bacteria. In a clonal population, cell-to-cell variation may result in phenotypic heterogeneity that is a mechanism to survive changing environments including antibiotic therapy. *Stenotrophomonas maltophilia* has been frequently isolated from cystic fibrosis patients, can cause numerous infections in other organs and tissues, and is difficult to treat due to antibiotic resistances. *S. maltophilia* K279a produces the L1 and L2 β-lactamases in response to β-lactam treatment. Here we report that the patient isolate *S. maltophilia* K279a diverges into cellular subpopulations with distinct but reversible morphotypes of small and big colonies when challenged with ampicillin. This observation is consistent with the formation of elongated chains of bacteria during exponential growth phase and the occurrence of mainly rod-shaped cells in liquid media. RNA-seq analysis of small versus big colonies revealed differential regulation of at least seven genes among the colony morphotypes. Among those, *bla*_L1_ and *bla*_L2_ were transcriptionally the most strongly upregulated genes. Promoter fusions of *bla*_L1_ and *bla*_L2_ genes indicated that expression of both genes is also subject to high levels of phenotypic heterogeneous expression on a single cell level. Additionally, the *comE* homolog was found to be differentially expressed in homogenously versus heterogeneously *bla*_L2_ expressing cells as identified by RNA-seq analysis. Overexpression of *comE* in *S. maltophilia* K279a reduced the level of cells that were in a *bla*_L2_-ON mode to 1% or lower. Taken together, our data provide strong evidence that *S. maltophilia* K279a populations develop phenotypic heterogeneity in an ampicillin challenged model. This cellular variability is triggered by regulation networks including *bla*_L1_, *bla*_L2_, and *comE*.

## Introduction

*Stenotrophomonas maltophilia* is a Gram-negative, non-fermentative bacterium, which is usually associated with the rhizosphere but can cause opportunistic infections of the respiratory tract in immunocompromised patients. In recent years, *S. maltophilia* has been frequently isolated from cystic fibrosis patient. As a nosocomial pathogen, it can also cause numerous infections in other organs and tissues, including bacteremia, endocarditis, pneumonia, peritonitis, cellulitis, and meningitis (Agger et al., 1986; Elting and Bodey, 1990; Nguyen and Muder, 1994; Gutierrez Rodero et al., 1996; Elsner et al., 1997; Al-Hilali et al., 2000). Treatment is often difficult because the microorganism is intrinsically resistant against many common antibiotics including β-lactams (Denton and Kerr, 1998; Crossman et al., 2008; Ryan et al., 2009; Brooke, 2012). The intrinsic resistance of *S. maltophilia* K279a (SMK279a) against β-lactam antibiotics is primarily due to the function of two β-lactam resistance genes, *bla*_L1_ (*smlt2667*) and *bla*_L2_ (*smlt3722*), (Saino et al., 1982; Walsh et al., 1994, 1997). The product of *bla*_L1_ is an Ambler class B Zn^+^-dependent metalloenzyme, and the product of *bla*_L2_ is an Ambler class A serine active site β-lactamase. Both enzymes are responsible for inactivation of a wide range of different β-lactam antibiotics (Walsh et al., 1994, 1997; Al Naiemi et al., 2006).

Expression of *bla*_L1_ and *bla*_L2_, which is controlled by the activities of several proteins including AmpG, NagZ, AmpD, and the transcriptional regulator AmpR (Cullmann and Dick, 1990; Avison et al., 2002; Gould et al., 2006; Hu et al., 2008; Okazaki and Avison, 2008), has been suggested to be induced by β-lactam antibiotics. Furthermore, the expression of β-lactam resistance genes is linked to cell wall biosynthesis, a complex dynamic process affected by multiple cellular processes such as growth phase, division cycle, quorum sensing, and cell stress (Typas et al., 2012; Zeng and Lin, 2013). In addition to its autoregulation of transcription, AmpR regulates the induction of chromosomal β-lactamases in Gram-negative bacteria (Balcewich et al., 2010). A homologous *ampR-bla*_L2_ module with a similar induction mechanism has been identified earlier in *S. maltophilia* (Lin et al., 2009). In this bacterium*, ampR* (*smlt3723*) is physically linked to *bla*_L2_, and it is assumed that AmpR can also regulate the expression of the unlinked *bla*_L1_ gene (Okazaki and Avison, 2008). In the absence of an inducer, AmpR maintains its inactive conformational state as a result of binding to effector molecules, i.e., UDP-MurNAc-oligopeptides. However, exposure of the bacteria to β-lactam antibiotics increases cytosolic accumulation of anhydro-MurNAc-oligopeptides, a cell wall degradation product, which can displace the AmpR-associated UDP-MurNAc-oligopeptides. This leads to a conformational change of AmpR and subsequent activation of *bla*_L1_ and *bla*_L2_ transcription (Dietz et al., 1997; Jacobs et al., 1997; Balcewich et al., 2010).

While it is well known that the genetic background determines the ability of a bacterial strain to overcome antibiotic stress, bacteria have developed additional mechanisms to successfully overcome antibiotic treatment. These mechanisms may include smaller genetic variations such as single nucleotide polymorphisms (SNPs) and non-genetic variations. Within this context, phase variations can also be the cause of antibiotic resistance and/or phenotype switching. They occur at a rate of 1:10,000 in a population and can be the result of different events (Henderson and Owen, 1999; Tipton et al., 2015; and references herein).

Non-genetic variations that exist within an isogenic population contribute to the survival and fitness of the population (Ackermann, 2015; Grote et al., 2015). In a clonal population, cell-to-cell variation may result in a measurable phenotype termed phenotypic heterogeneity (Kaern et al., 2005; Magdanova and Golyasnaya, 2013). Phenotypic heterogeneity via heterogeneous gene expression benefits the population through division of labor and bet-hedging in a homogeneous environment as represented by laboratory culture conditions. This has been demonstrated for a number of model organisms such as *Salmonella enterica* serovar Typhimurium, *Vibrio harveyi, Bacillus subtilis*, *Sinorhizobium fredii*, and others (Anetzberger et al., 2009; Deris et al., 2013; Mulder and Coombes, 2013; Grote et al., 2014; Wang et al., 2014). Therefore, phenotypic heterogeneity is a way of multicellularity in bacterial populations to enhance the ability to adapt to changing environments (Shapiro, 1998; Davidson and Surette, 2008) and, consequently needs to be better understood to optimize antibiotic treatment strategies.

It has been shown that some bacteria can survive an antibiotic treatment by stochastically entering a dormant persister state during vegetative growth. The persister cells form transiently antibiotic tolerant subpopulations susceptible to re-introduction of the antibiotic stress, indicating that non-genetic mechanisms are involved in this process (Lewis, 2007, 2010). Much research has been focused on the exploration of the molecular keys linked to the persister phenomenon. For a more detailed insight into this topic, we refer to the excellent reviews given in references (Lewis, 2010; Helaine and Kugelberg, 2014; Maisonneuve and Gerdes, 2014). The persister phenotype depends on various factors such as the level of signaling nucleotide (p)ppGpp, and various metabolic activities (Maisonneuve et al., 2013; Amato et al., 2014). Thereby it is well accepted that toxin–antitoxin (TA) systems play a key role in the regulatory network of persisters. These systems consist of a ‘toxin’, which is normally a stable protein that interferes with vital cellular functions and a cognate ‘antitoxin’, an unstable protein or RNA molecule, which regulates the toxin level. The most prominent example for a TA system controlling persistence is the *Escherichia coli hipAB* TA system. However, additional systems have been described and ranked in the order of their importance (Keren et al., 2004; Lewis, 2007; Wu et al., 2015).

Here we report phenotypic heterogeneity in SMK279a upon exposure to β-lactam antibiotics. SMK279a was isolated from a blood sample of a hospitalized patient and has a genome size of 4,851,126 bp (Crossman et al., 2008). In the presence of ampicillin, SMK279a cells showed heterogeneity in colony and cell morphology, which goes along with colony-specific patterns of differentially expressed genes, including expression of the β-lactam resistance genes *bla_L1_ and bla_L2_*. As demonstrated with reporter gene fusions, *bla_L1_ and bla_L2_* are subject to differential regulation even at the single cell level. Together with genome-wide sequences of cells from the different colony morphotypes, our data show that phenotypic heterogeneity in *S. maltophilia* K279a is a result of non-genetic variations in individual cells within isogenic populations.

## Materals And Methods

### Bacterial Strains, Plasmids, and Growth Conditions

All strains and plasmids used in the present study are described in **Table 1**. *S. maltophilia* and *E. coli* strains were routinely grown either in LB medium (10 g/l tryptone, 5 g/l yeast extract and 5 g/l NaCl) or on LB agar plates at 30 or 37°C. Ampicillin (100 μg/ml), gentamicin (10 μg/ml), kanamycin (25 μg/ml), chloramphenicol (60 μg/ml), norfloxacin (5 μg/ml), or tetracycline (50 μg/ml) was added to the media as required. For analyses of phenotypic colony heterogeneity, SMK279a was spread on LB agar plates without or with ampicillin (100 μg/ml) and incubated at 37°C for 48 h. Pre-cultures were grown in 5 ml test tubes at 37°C. For analysis of single cell expression, cultures were grown for 12 to 15 generations in LB broth supplemented with gentamicin (50 μg/ml) and without β-lactam inducer. The Minimal Inhibitory Concentration (MIC) for ampicillin was analyzed using the microdilution technique and found to be at a level of 254 μg/ml.

**Table 1:** Bacterial strains and plasmids used in this work.

Strain or construct	Description	Reference
***Escherichia coli* strains**		
HB101	F^-^, *hsd*S20(r_B_^-^m_B_^-^), *rec*A13, *ara*14, *pro*A2, *lac*Y1, *gal*K2,*rps*L20 (Sm^R^) *xyl*-5, *mtl*-1, *sup*E44, λ^-^,	Figurski and Helinski, 1979
SY327	Δ(*lac pro*) *argE*(*Am*) *recA56 rif ^R^ nalA*λ *pir*	Miller and Mekalanos, 1988
DH5α	F^-^ Φ80*lacZ*ΔM15 Δ(*lacZYA-argF*) U169 *recA1 endA1 hsdR17*(r_K_^-^ m_K_^+^) *phoA supE44 thi-1 gyrA96 relA1*λ^-^	Hanahan, 1983
***Stenotrophomonas maltophilia* strains**		
SMK279a	Clinical isolate from the blood of a cancer patient	Avison et al., 2000
SMK279aΔ*smlt1134*	SMK279a lacking the *smlt1134* gene coding for a putative DNA transport competence protein	This study
SMK279aΔ*smlt2851*Δ *smlt2852*	SMK279a lacking the *smlt2851* and *smlt2852* genes coding for putative transmembrane eﬄux proteins	This study
SMK279aΔ*smlt3723*	SMK279a lacking the *ampR* (*smlt3723*) gene; a putative HTH and LysR family transcriptional regulator	This study
SMK279aΔ*smlt0387*	SMK279a carrying a deletion in *smlt0387*	This study
SMK279a EM2	SMK279a carrying pBBR1MCS-5::P*bla*_L2_::*rfp*	This study
SMK279a EM3	SMK279a carrying pBBR1MCS-5::P*bla*_L1_::*rfp*	This study
SMK279a EM4	SMK279a carrying pBBR1MCS-5::P_less_::*rfp*	This study
SMK279a EM5	SMK279a carrying pBBR1MCS-5::P*bla*_L2_::*cfp*	This study
SMK279a EM6	SMK279a carrying pBBR1MCS-5::P*bla*_L2_::*yfp*	This study
SMK279a EM7	SMK279a carrying pBBR1MCS-5::P*bla*_L2_s::*rfp*	This study
SMK279a EM8	SMK279a carrying pBBR1MCS-5::P*bla*_L2_::*rfp::smlt1134*	This study
SMK279a EM9	SMK279a carrying pBBR1MCS-5::P*bla*_L2_::*rfp:: smlt2851*::*smlt2852*	This study
CF148	Clinical isolate from the respiratory tract of a cystic fibrosis patient	Jörg Steinmann (Universitätsklinikum Essen)
DSM-50170	Reference strain from the oropharyngeal region of a patient with cancer	Leibniz institute DSMZ
**Plasmids**		
pRK2013	Kan^R^; RK2-derived helper plasmid carrying the *tra* and *mob* genes for mobilization of plasmids containing *oriT*	Figurski and Helinski, 1979
pBBR1MCS-5	Broad host range vector, low copy, Gm^r^	Kovach et al., 1995
pBBR1MCS-5::*rfp*	pBBR1MCS-5 carrying the *rfp* gene in the MCS	This study
pBBR1MCS-5::P*bla*_L1_::*rfp*	P*bla*_L2_::*rfp* reporter fusion in pBBR1MCS-5	This study
pBBR1MCS-5::P*bla*_L2_::*rfp*	P*bla*_L1_::*rfp* reporter fusion in pBBR1MCS-5	This study
pBBR1MCS-5::P*bla*_L2_::*yfp*	P*bla*_L2_::*yfp* reporter fusion in pBBR1MCS-5	This study
pBBR1MCS-5::P*bla*_L2_::*cfp*	P*bla*_L2_::*cfp* reporter fusion in pBBR1MCS-5	This study
pBBR1MCS-5::P_less_::*rfp*	Promoterless *rfp* reporter fusion in pBBR1MCS-5	This study
pBBR1MCS-5::P*bla*_L2_s::*rfp*	P*bla*_L2_s::*rfp* reporter fusion constructed with the *ampR-bla*_L2_ intergenic region in pBBR1MCS-5	This study
pBBR1MCS-5:: P*bla*_L2_::*rfp::smlt1134*	*smlt1134* gene under its native promoter cloned into the pBBR1MCS-5::P*bla*_L2_::*rfp* reporter fusion	This study
pBBR1MCS-5::P*bla*_L2_::*rfp:: smlt2851*::*smlt2852*	Putative operon of *smlt2851* and *smlt2852* under its native promoter cloned into the pBBR1MCS-5::P*bla*_L2_::*rfp* reporter fusion	This study
pGPI-SceI-XCm	Cm^R^, Tp^R^; mobilizable suicide vector; carries the R6Kγ origin of replication, the I-SceI recognition site and a *xylE* reporter gene	Hamad et al., 2010
pDAI-SceI-SacB	Tet^R^; mobilizable broad host range plasmid; carries the gene for the I-SceI homing endonuclease and the *sacB* gene	Flannagan et al., 2008; Hamad et al., 2010
pUDK011	pGPI-SceI-XCm with a 705-bp *Sph*I/*Kpn*I insert of SMK279a containing the flanking region upstream of *smlt1134*	This study
pUDK012	pUDK011with a 741-bp *Kpn*I/*Xba*I insert of SMK279a containing the flanking region downstream of *smlt1134*	This study
pUDK014	pGPI-SceI-XCm with a 779-bp *Not*I/*Kpn*I insert of SMK279a containing the flanking region upstream of *smlt2852*	This study
pUDK015	pUDK014 with a 776-bp *Kpn*I/*Xba*I insert of SMK279a containing the flanking region downstream of *smlt2851*	This study
pUDK017	pGPI-SceI-XCm with a 761-bp *Not*I/*Kpn*I insert of SMK279a containing the flanking region upstream of *smlt3723*	This study
pUDK018	pUDK017 with a 660-bp *Kpn*I/*Xba*I insert of SMK279a containing the flanking region downstream of *smlt3723*	This study


### Scanning and Fluorescence Microscopy

Scanning electron microscopy (SEM) was performed as previously published (Krohn-Molt et al., 2013). Therefore, bacterial cells were grown overnight in 5 ml LB broth or on LB agar plates for 48 h. The cell suspension was centrifuged at 13,000 rpm for 2 min. The pellets or the colonies were resuspended in 500 μl sterile PBS-buffer, fixed in paraformaldehyde (1%) and glutaraldehyde (0.25%) and dehydrated gradually after successive immersions in ethanol solutions of increasing concentrations (30, 50, 70, 90, and 96%). Each rinsing and dehydrating step lasted 10 min. Finally, cells were dehydrated overnight in absolute ethanol (99.6%). The drying step was completed by drying pelletized cells at the critical point with the Balzers CPD 030 Critical Point Dryer. After coating samples with gold using an SCD 050 sputter coater (Bal-Tec), scanning electron micrographs were taken with a Leo 1525 (Zeiss, Germany).

Single cell analyses were evaluated as previously described (Grote et al., 2014). The phase-contrast and fluorescence images were recorded using a Zeiss AxioCam microscope with an MRm camera mounted on the fluorescence microscope (Zeiss Axio Imager.M2; Carl Zeiss AG, Oberkochen, Germany). For fluorescence imaging, the microscope was equipped with filter BP546/12 (red), the emission filter 605/75 (red), and a Zeiss Illuminator HXP 120 C. Phase-contrast and fluorescence images were obtained from the same area and matched using the Axio-Vision software (release 4.8). The fluorescence phenotypes of single cells were recorded by evaluating, in general, a minimum of 400 cells per biological sample. For each treatment, at least three independent samples were analyzed by visually inspecting the obtained images.

### Molecular Cloning and Generation of SMK279a Mutants

For construction of the reporter gene fusions, the genes *rfp* (DsRed2; Baird et al., 2000), *cfp* (mCerulean; Rizzo et al., 2004) and *yfp* (mVenus; Shaner et al., 2004) were fused to DNA fragments carrying the promoter of the *bla*_L1_ gene or the promoter and intergenic region of the *bla*_L2_ gene. The DNA fragments containing the promoter region were 318 bp for *bla*_L2_, the intergenic region 180 bp for *bla*_L2_ and 417 bp for *bla*_L1_, and were predicted using PromBase (Rangannan and Bansal, 2011). These cassettes were cloned into the broad host range vector pBBR1MCS-5 (Kovach et al., 1995). Constructs were verified by DNA sequencing. The recombinant plasmids were transferred to SMK279a by triparental mating, using *E. coli* HB101 carrying the plasmid pRK2013 as a helper strain (Figurski and Helinski, 1979).

Markerless SMK279a mutants were constructed using the pGPI-SceI/pDAI-SceI-SacB system originally developed for bacteria of the genus *Burkholderia* (Flannagan et al., 2008; Aubert et al., 2014). The bacterial strains and plasmids of the pGPI-SceI/pDAI-SceI-SacB system were a generous gift from Miguel A. Valvano (Center for Infection and Immunity, Queen’s University, Belfast, UK). The mutagenesis method is based on the I-SceI homing endonuclease system, which relies on two independent crossover events to integrate first a deletion plasmid with a I-SceI recognition site into the genome of the recipient and then resolve the co-integrate structure by a second homologous recombination event in the presence of the I-SceI endonuclease provided *in trans* on plasmid pDAI-SceI-SacB. The deletion plasmid was derived from the mobilizable suicide vector pGPI-SceI-XCm and contained upstream and downstream flanking regions of the gene to be deleted. All deletion plasmids were generated and maintained in *E. coli* SY327 (Miller and Mekalanos, 1988), which constitutively expresses the λ *pir* gene product for replication of plasmids containing the R6Kγ origin of replication such as pGPI-SceI-XCm. To construct the deletion plasmid pUDK012 targeting the *smlt1134* gene of SMK279a, the primers KOsmlt1134-1, and KOsmlt1134-2 (**Table 2**) were used to amplify a 705 bp fragment of the flanking region upstream of *smlt1134*, followed by digestion of the PCR product with *Sph*I/*Kpn*I and cloning into the *Sph*I/*Kpn*I sites of pGPI-SceI-XCm. The resulting plasmid was designated pUDK011. The flanking downstream region of *smlt1134* of 741 bp was obtained by PCR with primer pair KOsmlt1134-3/KOsmlt1134-4. The PCR product was then digested with *Kpn*I/*Xba*I and cloned into the *Kpn*I/*Xba*I sites of pUDK011 to yield pUDK012. For deletion of the genomic region containing the *smlt2852* and *smlt2851* genes, the plasmid pUDK015 was constructed. As a first step, the plasmid pUDK014 was generated by amplification of the flanking upstream region of *smlt2852* with primers KOsmlt2852-1 and KOsmlt2852-2, digestion of the PCR product with *Not*I/*Kpn*I and ligation of the 779 bp insert into the *Not*I/*Kpn*I sites of pGPI-SceI-XCm. The primer pair KOsmlt2851-3/KOsmlt2851-4 was subsequently used to amplify a 776 bp fragment containing the flanking downstream sequences of *smlt2851*, followed by cloning of the *Kpn*I- and *Xba*I-digested PCR product into the *Kpn*I/*Xba*I sites of pUDK014 to yield the deletion plasmid pUDK015.

**Table 2:** Primers used in this study.

Primer	Sequence [5′-3′]
Smlt3722For	CTTAGGTACCCGGATCTGGTGGCTCAGT
Smlt3722Rev	CGATGAATTCCGAGCATGCGGGTTCTCCTG
3722rfpFor	CTTAGGTACCCATCGCGCAGTCGTGA
3722rfpRev	CGTTGAATTCATGCGGGTTCTCCTGG
Ma2667For	CTTAGGTACCAACCGGCTGACTGCGTTCT
Ma2667Rev	CGATGAATTCGATCCACGTCCGCTTGAAG
KOsmlt1134-1	ATATTgcatgcCTCTTCACAGGCTTCGATCAGC*^a^*
KOsmlt1134-2	CTAGACggtaccCAAGGCACAACGTGTATCTCCC*^b^*
KOsmlt1134-3	CCCGGATCggtaccGGTGCG*^b^*
KOsmlt1134-4	CTTCTtctagaTGTTGTAGAGGTTGGACCTGTGG*^c^*
KOsmlt2852-1	CCTGAgcggccgcCACGTACACCGACAGTTCG*^d^*
KOsmlt2852-2	TAGACggtaccATGGGCCATGAACGAGGTG*^b^*
KOsmlt2851-3	CATGCggtaccGCATCGCATTGGTGACCGTC*^b^*
KOsmlt2851-4	TACCGtctagaGCTTCGATTCCAGCAACTGGG*^c^*
KOsmlt3723-1	CCTGAgcggccgcTTGGCAAAGCTGTTCAGCTCC*^d^*
KOsmlt3723-2	TAGACggtaccTTCAGTGGCAGGGTAGGGTG*^b^*
KOsmlt3723-3	CATGCggtaccGCATGTTCGAGCAGGAACTGG*^b^*
KOsmlt3723-4	TACCGtctagaGATCGTGATGGTCTCTCACGAC*^c^*


**^a^Sph*I site is shown in lower case letters.*

**^b^Kpn*I site is shown in lower case letters.*

*^c^XbaI site is shown in lower case letters.*

*^d^NotI site is shown in lower case letters.*

The plasmid pUDK018 for deletion of the *smlt3723* gene was generated in a similar way. Using the primers KOsmlt3723-1 and KOsmlt3723-2, a 761 bp fragment homologous to the flanking region upstream of *smlt3723* was amplified. The PCR product was then digested with *Not*I and *Kpn*I for ligation into the *Not*I/*Kpn*I sites of pGPI-SceI-XCm. As a result, the plasmid pUDK017 was obtained. Finally, the deletion plasmid pUDK018 was derived from pUDK017 by amplification of a 660 bp fragment containing the flanking region downstream of *smlt3723* with primers KOsmlt3723-3 and KOsmlt3723-4, and cloning of the *Kpn*I/*Xba*I-digested PCR product into the *Kpn*I/*Xba*I sites of pUDK017.

The successful construction of all plasmids was verified by DNA sequence analysis of the inserts. The deletion plasmids were transferred to SMK279a by triparental mating as described previously (Aubert et al., 2014), using *E. coli* DH5α carrying the plasmid pRK2013 as a helper strain, and *E. coli* SY327/pUDK012, SY327/pUDK015, and SY327/pUDK018 as the donor strains, respectively. Selection for SMK279a co-integrants was performed at 37°C on LB agar plates containing 60 μg/ml chloramphenicol and 5 μg/ml norfloxacin to counter-select against the *E. coli* helper and donor strains. Streaks of SMK279a exconjugants were subsequently sprayed with 0.45 M pyrocatechol to confirm integration of the deletion plasmids into the genomes of co-integrants. Due to the activity of the pyrocatechol 2,3-dioxygenase encoded by the *xylE* reporter gene of the deletion plasmids, a bright yellow color of the biomass was indicative of the presence of pUDK012, pUDK015, and pUDK018 in the genomes of the strains, respectively.

As a next step, the plasmid pDAI-SceI-SacB was introduced into the SMK279a co-integrants by triparental mating with DH5α/pRK2013 and DH5α/pDAI-SceI-SacB as helper and donor strains, respectively. Exconjugants were selected at 30°C on LB agar plates containing 50 μg/ml tetracycline and 5 μg/ml norfloxacin, followed by screening for excision of the deletion plasmids. Both the inability of pyrocatechol to turn the color of the exconjugants into bright yellow and sensitivity of the strains to 60 μg/ml chloramphenicol indicated the loss of the integrated plasmids. Finally, the plasmid pDAI-SceI-SacB was cured by sucrose counter-selection as described (Aubert et al., 2014). As a result, the unmarked mutants SMK279aΔ*smlt1134*, SMK279aΔ*smlt2851*Δ*smlt2852* and SMK279aΔ*smlt3723* were obtained. The deleted regions of the mutant strains were verified by PCR and DNA sequence analysis.

The mutant strain SMK279aΔ*smlt0387* was generated according to Hoang et al. (1998) and Alavi et al. (2013).

### RNA-seq and Data Analysis

To prepare the cell material for RNA-seq analyses, SMK279a was grown on LB agar plates with/without ampicillin (100 μg/ml). Two samples from each colony morphotype (small and big colonies) and two samples from uniform colonies were scrapped from agar plates after 48 h of growth at 37°C, pooled and immediately frozen in liquid nitrogen. About 10–15 mg of wet biomass were generally used for each biological replicate, 10 mg of cell material corresponded to an OD_600_ of 6.35. Additionally, for RNA extraction of liquid cultures, the strain SMK279a carrying the P*bla*_L2_::*rfp* reporter fusion was grown for 27 and 32 h with 100 μg/ml ampicillin. Total RNA was extracted using the hot-phenol method as described previously (Blomberg et al., 1990). The residual genomic DNA was removed from the total isolated RNA by DNase I treatment. All samples were adjusted to contain equal amounts of extracted RNAs. The cDNA libraries for sequencing were constructed by vertis Biotechnology AG, Germany as described by Sharma et al. (2010). The transcripts were not fragmented. The cDNA libraries were sequenced using a HiSeq 2500 machine (Illumina) in single-read mode and running 100 cycles. To assure a high sequence quality, the Illumina reads in FASTQ format were trimmed with a cut-off phred score of 20 by the program fastq_quality_trimmer from FASTX toolkit version 0.0.13^1^. The alignment of reads, coverage calculation, gene wise read quantification and differential gene expression was performed with READemption (Förstner et al., 2014) version 0.3.5 (doi: 10.5281/zenodo.13926), ‘segemehl’ (Hoffmann et al., 2009) version 0.2.0 and DESeq2 (Love et al., 2014) version 1.6.3. Visual inspection of the coverages was done using the Integrated Genome Browser (IGB; Nicol et al., 2009). The reference sequence for SMK279a was retrieved from the NCBI database (accession no.: NC_010943.1). Genes with a fold-change of ≥2.0 and an adjusted *P*-value (corrected for multiple testing using the Benjamini–Hochberg procedure) ≤0.05 were considered as differentially expressed. The raw, de-multiplexed reads as well as coverage files have been deposited in the National Center for Biotechnology Information’s Gene Expression Omnibus under the project ID: GSE71735. A shell script that covers the main RNA-Seq data processing steps was deposited at https://zenodo.org/record/34153 (DOI:10.5281/zenodo.34153).

### RT-qPCR

Selected differentially regulated genes identified in SMK279a RNA-seq analyses were verified by RT-qPCR experiments as described previously (Krysciak et al., 2014). Gene-specific primers used for RT-qPCR are shown in **Table 3**. Samples for RNA extraction were taken from colonies that had been grown for 48 h in the presence of ampicillin (100 μg/ml) and were obtained from three independent experiments. The SuperScript^®^ VILO^TM^ cDNA synthesis kit (Invitrogen^TM^, life technologies, TX, USA) was used to generate cDNA using 1.2 μg RNA. The expression values were normalized against *rpoD* and 16S rRNA.

**Table 3:** Gene-specific primers used for RT-qPCR.

Primer	Sequence [5′-3′]	Length of amplified product/gene
qsmlt4165-Bfor	AAGGGCCTGGAACAGGGCTA	145 bp/*rpoD*^∗^
qsmlt4165-Brev	CCACATCCGGCGCAACTTCA	
q16S-Afor	AGAGTTTGATCCTGGCTCAG	240 bp/16S rRNA^∗^
q16S-Arev	CTAATCCGACATCGGCTCAT	
qsmlt2667-for	CGGTCACCTGCTGGACAACA	157 bp/*bla*_L1_
qsmlt2667-rev	CACCGCCGTTTCTGCATTGG	
qsmlt3722-Afor	CGTCGCCGATTCCTGCAGTT	144 bp/*bla*_L2_
qsmlt3722-Arev	TTCCAGCGCGGCGAAATCAC	
qsmlt3721-for	GAGCCATGAACTGATCTACC	196 bp/*smlt3721*
qsmlt3721-rev	GAACAGGAACAGCGAGGAAA	
qsmlt0018-for	CGGAACTGGAGTTCACTCTT	168 bp/*smlt0018*
qsmlt0018-rev	TGATGTCCCAATCCGTTAGC	
qsmlt0019-for	TAATACTAACGGCCCGACCT	205 bp/*smlt0019*
qsmlt0019-rev	TCTTCCTGCACCTGAGTTCT	
qsmlt1007-for	GAAGGCTTCACCAAGCTCAC	126 bp/*smlt1007*
qsmlt1007-rev	GATGCCGGTCCAGATCGTAT	


*^∗^Genes used for normalization of gene expression.*

### Whole Genome Sequencing and Variant Detection

Usually a single colony was transferred to fresh LB and cultured overnight to an OD_600_ of 1.0. Cells were pelleted, and the DNA was isolated with the AquaPure Genomic DNA Kit (Bio-Rad, Hercules, CA, USA). Libraries were generated following the Nextera XT DNA Library Preparation Kit (Illumina, San Diego, CA, USA) and sequenced on the MiSeq (2 × 310 bp) or NextSeq 500 (2 × 151 bp) platform (Illumina, San Diego, CA, USA). Respective FASTQ files were submitted to the EMBL EBI ENA short read archive^2^ (accession no.: ERP011093). Resulting reads were mapped to the SMK279a genome sequence (accession no.: NC_010943.1) with the alignment program SARUMAN (Blom et al., 2011). All isolates were sequenced with a minimum coverage of 50-fold. For variant detection in mapped reads, we employed customized Perl scripts using a minimum threshold of 10-fold coverage and a minimum allele frequency of 75% (Roetzer et al., 2013). Variant positions were then combined, supplementing the joint list with the respective information from the original mappings. Concatenating all SNP positions with a reliable base call (10-fold coverage and 75% allele frequency) in at least 95% of the isolates and no other SNP within a distance of 12 bp in the same strain yielded a sequence alignment for the construction of a maximum parsimony tree, which was built with the software BioNumerics version 7.5 (Applied Maths, Sint-Martens-Latem, Belgium).

## Results

### Transient Colony Heterogeneity and Formation of Outer Membrane Vesicles in Response to Antibiotic Treatment

**FIGURE 1:**
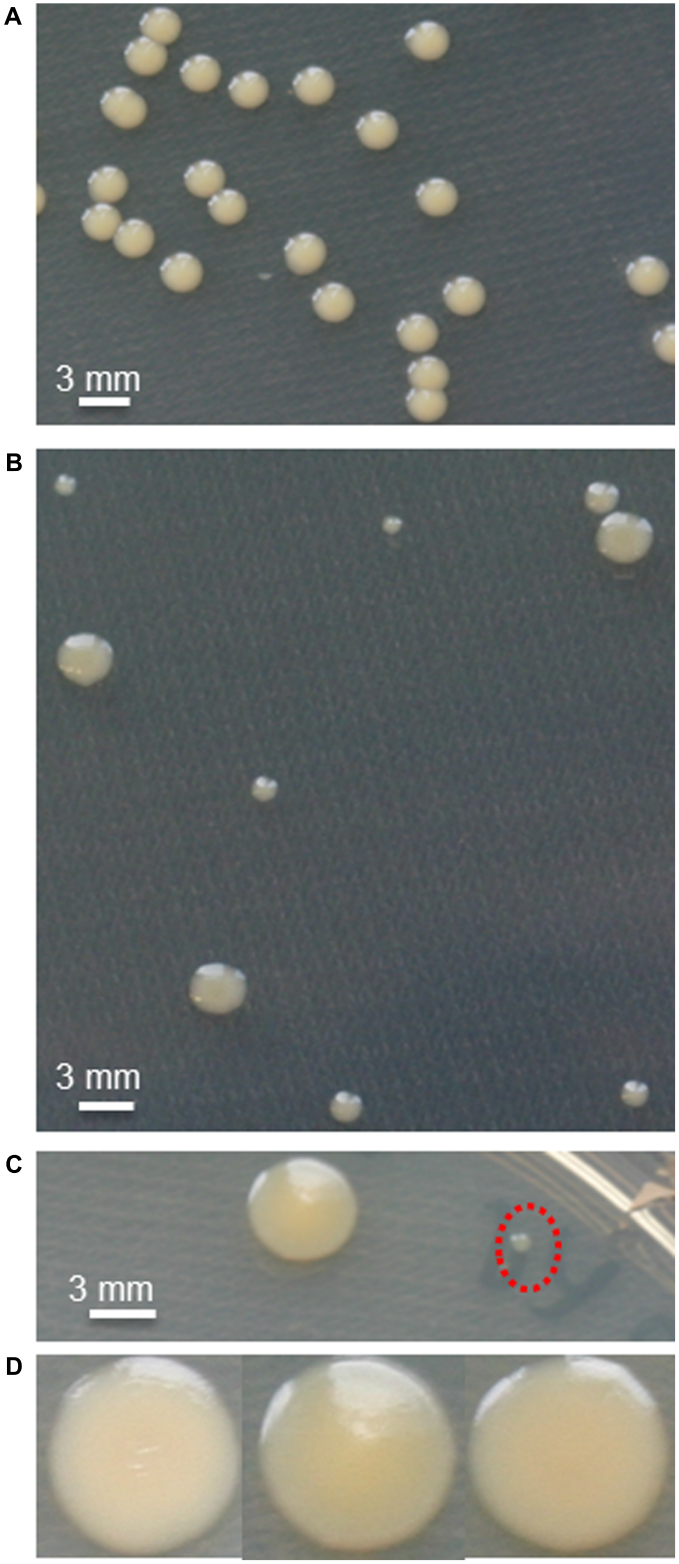
**Phenotypic heterogeneity of SMK279a cells during growth on solid media.** **(A)** SMK279a forms round, uniform colonies when grown without ampicillin on LB agar plates for 48 h at 37°C. **(B)** Colony heterogeneity on LB agar supplemented with ampicillin showing small and big colonies after growth for 48 h at 37°C. **(C)** Small colonies (indicated by red dashed circle) and big colonies cultivated on agar plates containing high concentrations of ampicillin (600 μg/ml). **(D)** Colonies differ slightly in color when cultivated for 48 h on agar plates containing high concentrations of ampicillin (600 μg/ml).

In the absence of antibiotics, the SMK279a wild-type strain forms cream-colored, round and uniform colonies with a diameter of approximately 1.75–2.0 mm after 24 h of growth on LB agar plates at 37°C. Addition of ampicillin (100 μg/ml) resulted in transient colony heterogeneity, with colonies differing in size and appearance (**Figure 1**) and could be observed as soon as they were visible on the plates. This was usually after 16–18 h of growth at 37°C for big colonies. Small colonies appeared between 24 and 33 h of growth. Colonies were grouped into three categories based on size and origin, i.e., small and big colonies in the presence of ampicillin (**Figure 1B**) and uniform colonies (**Figure 1A**) in the absence of ampicillin (**Table 4**). Formation of different colony morphotypes was independent of the presence or absence of ampicillin in the pre-cultures. When grown in the presence of high concentrations of ampicillin (600 μg/ml), small and big colonies were observed (**Figure 1C**) and, furthermore, colonies slightly changed the color and appearance (**Figure 1D**). Among these colonies, some were pointed at the center while others appeared to have a flat colony surface with a wrinkled texture.

**Table 4:** SMK279a colony morphotypes observed after 48 h of growth on LB agar plates with and without ampicillin.

Treatment	Concentration [μg/ml]	Colony morphotype	% of all colonies	Colony size [diameter, mm]
Ampicillin	100	Small	80	1.4 ± 0.26
Ampicillin	100	Big	20	3.2 ± 0.27
No ampicillin	–	Uniform	100	3.0 ± 0.06


When we streaked small colonies onto fresh plates without antibiotics, they formed uniform large colonies after overnight incubation, indicating the small colony morphotype was not caused by a genetic mutation. More importantly, small colonies formed both big and small colonies when recultivated in the presence of ampicillin. However, in the presence of ampicillin, cells from big colonies formed again a uniform colony at 24 and 48 h after transfer to fresh agar plates containing ampicillin. This reversible variation in colony morphology was also observed for other strains such as *S. maltophilia* CF148, an isolate from the respiratory tract of a cystic fibrosis patient, and *S. maltophilia* DSM-50170 isolated from the human oropharyngeal region (**Table 1**).

**FIGURE 2:**
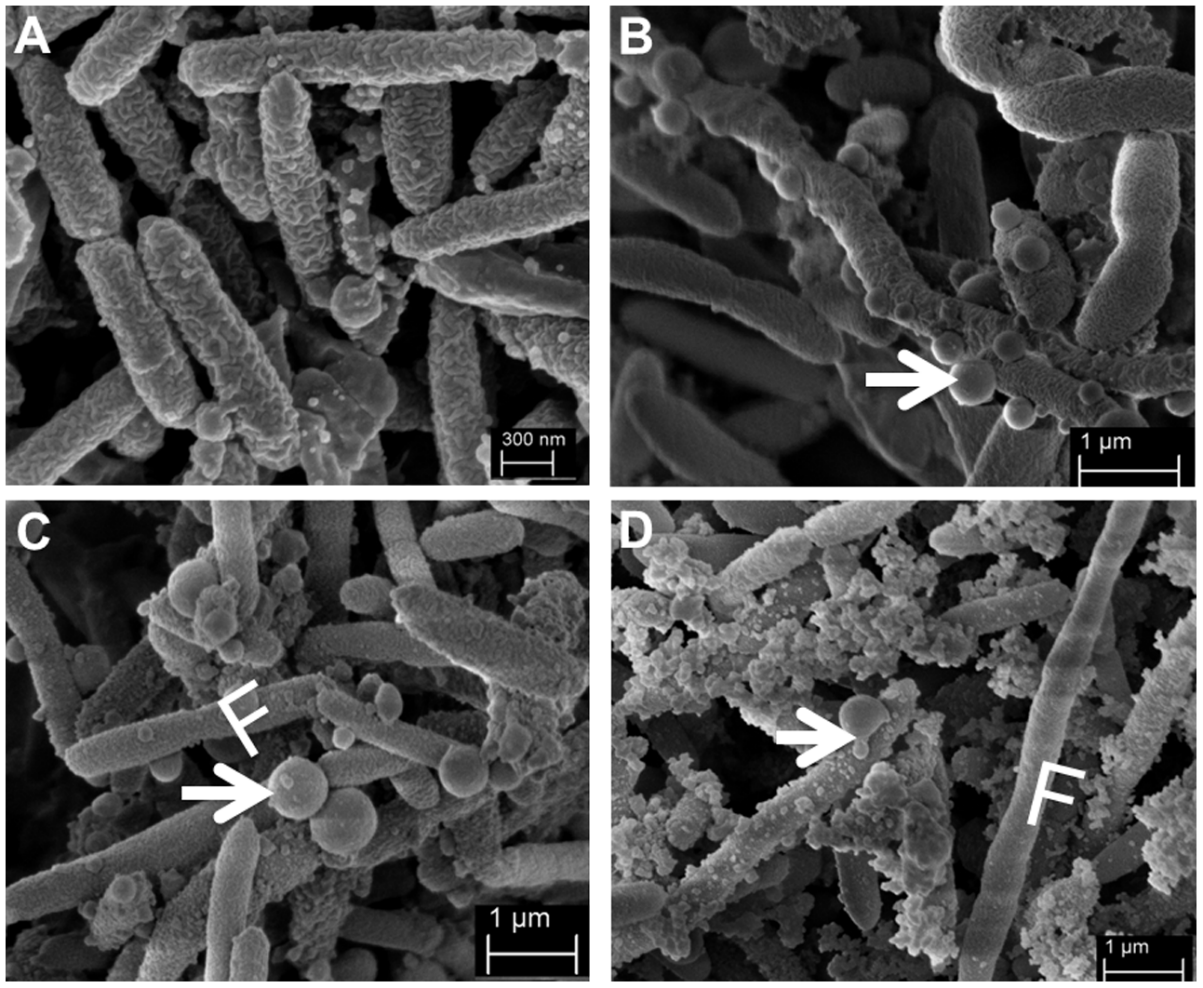
**Representative scanning electron micrographs (SEM) of SMK279a cells grown in the presence or absence of ampicillin at 37°C for 48 h on LB agar plates.** SEM images of SMK279a cells were recorded as previously published (Krohn-Molt et al., 2013). **(A)** SEM image of cells obtained from colonies cultured in the absence of ampicillin. **(B)** SEM image of cells obtained from small colonies cultured in the presence of 100 μg/ml ampicillin. **(C)** SEM image of cells obtained from big colonies cultured in the presence of 100 μg/ml ampicillin. **(D)** SEM image of cells obtained from big colonies cultured in the presence of 300 μg/ml ampicillin. Cells from **(A–C)** predominantly formed long filamentous cells (indicated by letter F) and OMVs (indicated by arrows). In the presence of ampicillin, SMK279a cells became enlarged and formed OMVs with sizes up to 677 nm in diameter.

Together with these findings, we observed that cells grown in the presence of ampicillin formed more frequently long bacterial chains (**Figure 2**). Furthermore, SMK279a cells from both colony morphotypes showed the formation of outer membrane vesicles (OMVs). However, this feature was much more pronounced in the presence of ampicillin (**Figures 2B–D**) in comparison to cells grown in the absence of the antibiotic that showed only very few or no vesicles (**Figure 2A**). Intriguingly, the size of the OMVs varied considerably, ranging from less than 100 nm up to 677 nm in diameter in some SMK279a cells, which was twice the size as those reported for other Gram-negative bacteria (Beveridge, 1999). Most likely, these vesicles are packed with *bla*_L1_ and *bla*_L2_ active enzymes (Devos et al., 2015).

These initial data suggested that the observed colony morphotypes resulted either from gene expression differences at the cellular level within isogenic populations or from heterogeneous expression of resistance genes at a single cell level. Since colony morphotypes were reversible, they most likely were not a result of permanent mutations such as SNPs. To test this hypothesis, we sequenced DNA from 24 individual colonies with diverse morphotypes using NGS technology (**Supplementary Figure S1**). Overall, we analyzed the genomes of 13 small, 9 big, and 2 uniform colonies with an average 51.4-fold coverage. Compared with the published SMK279a genome sequence, we identified 2–15 SNPs per colony, up to seven deletions and four insertions within all colonies (**Supplementary Table S1**). Most remarkably, none of these were identified within the known resistome of SMK279a, further supporting the notion that the colony morphotypes were not a primary result of genetic alterations.

A more detailed genome analysis identified SNPs or smaller deletions in 15 genes and ORFs, and none of these appeared to be linked to a gene that is essential for growth (**Supplementary Table S2**). Only one of the observed SNPs may be linked to β-lactam treatment, namely *smlt3885*, a gene which codes for a putative UDP-*N*-acetylmuramate:L-alanyl-gamma-D-glutamyl-meso-diaminopimelate ligase. This enzyme is most likely involved in recycling of cell wall precursors during bacterial cell wall synthesis but is not essential for growth (Mengin-Lecreulx et al., 1996). Two other regions in the SMK279a genome contained high densities of SNPs and indels. These loci are assigned to genes coding for the hypothetical protein Smlt1844B and a two-component regulatory system-sensor histidine kinase (Smlt3944). The *smlt1844B* and *smlt3944* genes carried non-synonymous mutations at seven or nine base positions, respectively (**Supplementary Table S2**). The product of *smlt3944* has a length of 880 amino acids and may be transcribed within an operon with the *smlt3943* gene coding for the phosphate-selective porin OprP (**Supplementary Figure S2**). OprP is mainly expressed under conditions of phosphate starvation (Siehnel et al., 1992), and with the flanking gene *smlt3942* (encoding a C4-dicarboxylate transport protein), it is presumably under the regulatory effect of this sensor histidine kinase (Yurgel and Kahn, 2004).

Based on these findings, we concluded that the observed colony heterogeneity is most likely due to reversible genetic switches causing differential gene expression at either a population and/or single cell level.

### Differential Gene Expression in Small Versus Big Colonies

The observed colony heterogeneity and differences in cell morphology prompted us to ask the question as to what extent the colony morphotypes would show different transcription profiles at a genome wide level. Therefore, we isolated total RNA from small, big and uniform colonies that were grown at 37°C for 48 h on LB plates. For this purpose, the different colonies were scraped off from the agar plates and pooled to obtain 10–15 mg of cell material for each colony type. We used colonies for RNA extraction as we expected that gene expression profile might reflect a different expression level given the different phenotypes. Given the above observation that the small colonies needed 24–33 h to appear on plates, a harvest at 48 h ensured us sufficient biomass and highly reproducible colony phenotypes for the RNA extraction.

After RNA extraction, cDNA libraries were constructed and sequenced using NGS. For each colony variant, we analyzed two independent biological replicates. Between 7.35 and 12.35 million reads were generated for the two biological replicates and aligned to the reference genome sequence of SMK279a (GenBank: NC_010943.1). The metadata of the samples and RNA-seq analyses are summarized in **Table 5**.

**Table 5:** Overall transcriptome metadata for the analyzed SMK279a colony morphotypes and liquid culture samples.

Sample no.	Sample type	Sample time point	No. of reads generated (×10^6^)	No. of uniquely mapped reads (×10^6^)
1	Big colony	48 h	8.80	0.69
2	Big colony	48 h	9.88	0.80
3	Small colony	48 h	9.72	0.63
4	Small colony	48 h	12.35	0.98
5	Uniform colony	48 h	7.35	0.38
6	Uniform colony	48 h	9.60	1.25
7	Liquid culture	27 h	6.30	0.39
8	Liquid culture	27 h	6.90	0.31
9	Liquid culture	32 h	6.84	0.46
10	Liquid culture	32 h	6.85	0.33


**FIGURE 3:**
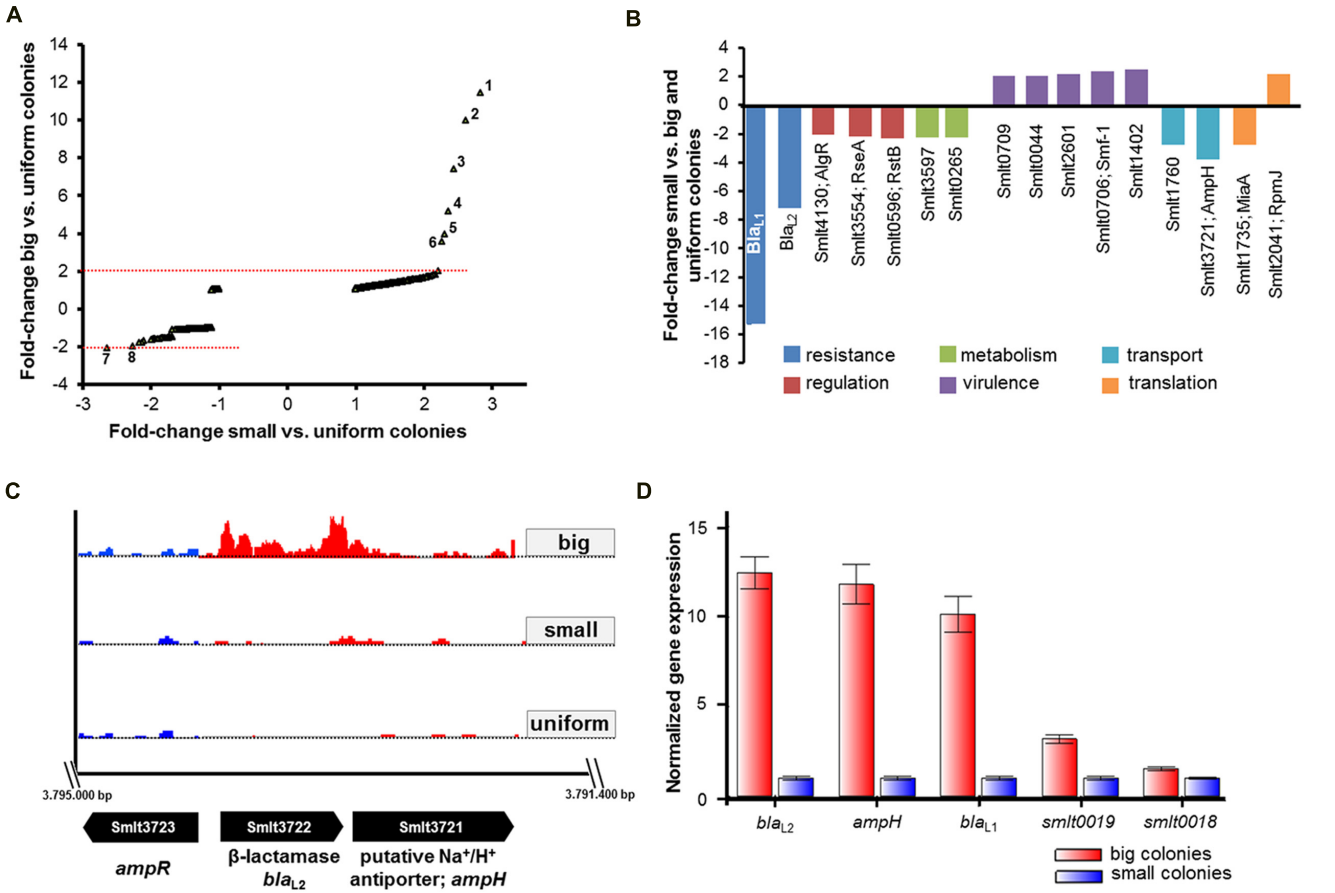
**RNA-seq and RT-qPCR data analysis for different SMK279a colony morphotypes.** **(A)** Scatter plot of fold-change in transcription of genes among cells of big (*Y*-axis) and small (*X*-axis) colonies relative to untreated samples of uniform colonies grown in the absence of ampicillin on LB agar plates. A twofold change in the transcription of genes with adjusted *P*-value of ≤0.05 is considered as significantly, differentially regulated. Cells forming big colonies differentially regulated several genes mainly involved in degradation of antibiotics. The numbers in the plot indicate most strongly regulated genes: 1, *smlt0018*; 2, *smlt2667*; 3, *smlt3772*; 4, *smlt2668*; 5, *smlt0019*; 6, *smlt3721*; 7, *smlt1760*; and 7, *smlt0596*. **(B)** Differentially regulated genes in small colonies in comparison to big and uniform colonies. Down-regulation of *bla*_L1_ and *bla*_L2_ affected genes involved in regulation, metabolism, virulence, transport and genetic information processing/translation (for details see **Table 6**). **(C)** Transcriptome profiles of *bla*_L2_ and the flanking genes *ampR* (*smlt3723*) and *ampH* (*smlt3721*) among the colony morphotypes. The *bla*_L2_ gene was 6.9-fold and 7.4-fold down-regulated in cells forming small colonies in comparison to big and uniform colonies, respectively. Transcriptome profile images of the leading strand (indicated in red) and the lagging strand (indicated in blue) were generated with the IGB software (Nicol et al., 2009), merged and rearranged on the leading strand for a simplified visualization. **(D)** RT-qPCR analysis of genes, which were identified in the RNA-seq data set. Expression profiles of five different genes (*bla*_L2_; *ampH*; *bla*_L1_; *smlt0019*; *smlt0018*) were obtained from three independent experiments and analyzed based on the normalized gene expression [2^-ΔΔ^*^C^*^(t)^ method], using *rpo*D and 16S rRNA as internal reference genes.

For comparative analyses of RNA-seq data, we considered genes with a fold-change of ≥2.0, a Likelihood value ≥0.9 and an adjusted *P*-value of ≤0.05 as statistically significant and differentially expressed between the colony variants. The RNA-seq data unveiled that isogenic populations of SMK279a cells differentially regulate only a small number of genes between the colony morphotypes. Surprisingly, only 12 genes were significantly and differentially regulated between big and small colony variants (**Table 6**, **Figure 3A**). Among the differentially expressed genes, *bla*_L1_ (*smlt2667*) and *bla*_L2_ (*smlt3722*) were 15.3- and 6.9-fold up-regulated in cells forming big colonies in comparison to those forming small colonies, respectively (**Table 6**, **Figure 3B**). Upon β-lactam challenge, the transcriptional response in a small colony morphotype further included an altered expression level of genes involved in diverse cellular functions. The level of altered expression of these genes ranged from 2.0 to 3.6-fold, including down-regulation of eight of 14 genes, and up-regulation of six out of the 14 regulated genes (**Figure 3B**).

**Table 6:** Differentially expressed genes for three colony morphotypes in SMK279a.

Locus tag	Predicted function	Fold-change big vs. small	Fold-change big vs. uniform	Fold-change small vs. uniform
Smlt0018	Hypothetical protein	+7.6	+11.4	–
Smlt0019	Hypothetical protein	+2.2	+3.9	–
Smlt0706	Fimbria adhesin protein Smf-1	-2.3	-2.0	–
Smlt0709	Fimbria adhesin protein	-2.0	–	–
Smlt1735	tRNA delta(2)-isopentenyl pyrophosphate MiaA	+2.4	–	–
Smlt1766	Hypothetical protein	+2.1	–	–
Smlt2667	β-lactamase Bla_L1_	+15.3	+9.9	–
Smlt2668	Hypothetical protein	+5.9	+5.1	–
Smlt3721	Putative Na^+^/H^+^ antiporter AmpH	+3.6	+3.5	–
Smlt3722	β-lactamase Bla_L2_	+6.9	+7.4	–
Smlt1844	Modification methylase	-2.0	–	–
Smlt2601	Poly (beta-D-mannuronate) lyase	-2.1	–	–
Smlt0003	Hypothetical protein	–	–	+2.1
Smlt0028	DNA helicase II	–	–	+2.0
Smlt0044	RHS-repeat-containing protein	–	–	+2.0
Smlt0201	Hypothetical protein	–	–	+2.2
Smlt0265	Acyl CoA dehydrogenase	–	–	-2.1
Smlt0336	Hypothetical protein	–	–	+2.3
Smlt0353	Transposase	–		+2.0
Smlt0596	Sensor histidine kinase RstB	–	–	-2.2
Smlt0641	Undecaprenyl-phosphate 4-deoxy-4-formamido-l-arabinose transferase	–	–	+2.4
Smlt1064	Hypothetical protein	–	–	+2.8
Smlt1391	Pseudogene	–	–	+2.1
Smlt1402	Methyl-accepting chemotaxis protein	–	–	+2.0
Smlt1664	Hypothetical protein	–	–	+2.6
Smlt1760	Major facilitator super family transmembrane protein	–	-	-2.6
Smlt2041	50S ribosomal protein L36 RpmJ	–	–	+2.0
Smlt3041	Hypothetical protein	–	–	+2.3
Smlt3056	Transmembrane protein	–	–	+2.1
Smlt3554	Anti-sigma factor RseA	–	–	-2.1
Smlt3597	Dehydrogenase	–		-2.1
Smlt4130	Two-component system response regulator AlgR	–	–	-2.0
Smlt4447A	Pseudogene	–	–	+2.2
Smlt4604	Endonuclease L-PSP family protein	–	–	+2.0


*The table shows genes with a fold-change of ≥2.0, a Likelihood value ≥0.9 and an adjusted *P-*value of ≤0.05.*

Moreover, as summarized in **Table 6**, 34 genes were identified to be differentially regulated in small colonies when compared to big and uniform colonies. Thus, four hypothetical proteins (Smlt0018, Smlt0019, Smlt1766, and Smlt2668), a putative Na+/H+ antiporter (Smlt3721, AmpH) and two fimbriae adhesin proteins (Smlt0706, Smf-1, and Smlt0709) are associated with the formation of the different colony morphotype. The fimbrial operon includes *smf-1* and *smlt0709* and is involved in haemagglutination, biofilm formation, and adherence to cultured mammalian cells (de Oliveira-Garcia et al., 2003). Notably, *smlt0018* and *smlt0019* most likely form an operon. Furthermore, although *ampH* is physically linked to *bla*_L2_ and the flanking *ampR* gene (*smlt3723*; **Figure 3C**), the function of *ampH* remains to be determined.

Expression analysis by RT-qPCR was used to confirm the RNA-seq data. We analyzed the expression profiles of five different genes (*bla*_L2_; *ampH*; *bla*_L1_; *smlt0019*; *smlt0018*) that were differentially transcribed between cells obtained from big and small colonies, including *rpo*D and 16S rRNA as internal reference genes. We analyzed expression data based on the normalized gene expression (2^-ΔΔ^*^C^*^T^ method), and the results confirmed the data obtained by RNA-seq (**Figure 3D**). The β-lactamase genes *bla*_L1_ and *bla*_L2_, as well as the putative Na^+^/H^+^ antiporter gene *ampH* were among the strongly upregulated genes in cells forming big colonies.

Since AmpR is the central regulator for the expression of β-lactamases, we analyzed the basal expression of *ampR* (*smlt3723*) in big vs. small colony variants. Therefore, we constructed an *ampR*-deletion mutant. The SMK279aΔ*smlt3723* deletion was verified by PCR and whole genome sequencing. Although the SMK279aΔ*smlt3723* mutant failed to grow on LB agar plates in the presence of 100 μg/ml ampicillin, it grew well in the absence of any added antibiotic. Therefore, this deletion mutant could not be used to analyze colony morphotypes in the presence of ampicillin.

### Heterogeneous Expression of *bla*_L1_ and *bla*_L2_ in Single Cells

In addition to the observed phenotypic heterogeneity with respect to colony morphologies and OMV formation, we speculated that β-lactamase expression might be subject to heterogeneous expression at a single cell level. To address this hypothesis, we constructed different reporter fusions in the broad host range vector pBBR1MCS-5 (Kovach et al., 1995), using the promoter regions of *bla*_L1_ or *bla*_L2_ fused to the red (RFP), yellow (YFP), and cyan (CFP) fluorescent proteins (**Table 1**). All obtained constructs were verified by sequencing and introduced into SMK279a by tri-parental mating using *E. coli* HB101 with pRK2013 as a helper plasmid (Figurski and Helinski, 1979). In the presence of ampicillin, SMK279a cells grew in long chains during the exponential growth phase rather than in individual rods or aggregates as observed during stationary growth phase. Furthermore, the ampicillin susceptibility of this bacterium was unchanged indicating that the native AmpR binding ligand remained intact and was not affected *in trans* by the P*bla*_L2_::*rfp* promoter construct (data not shown).

**FIGURE 4:**
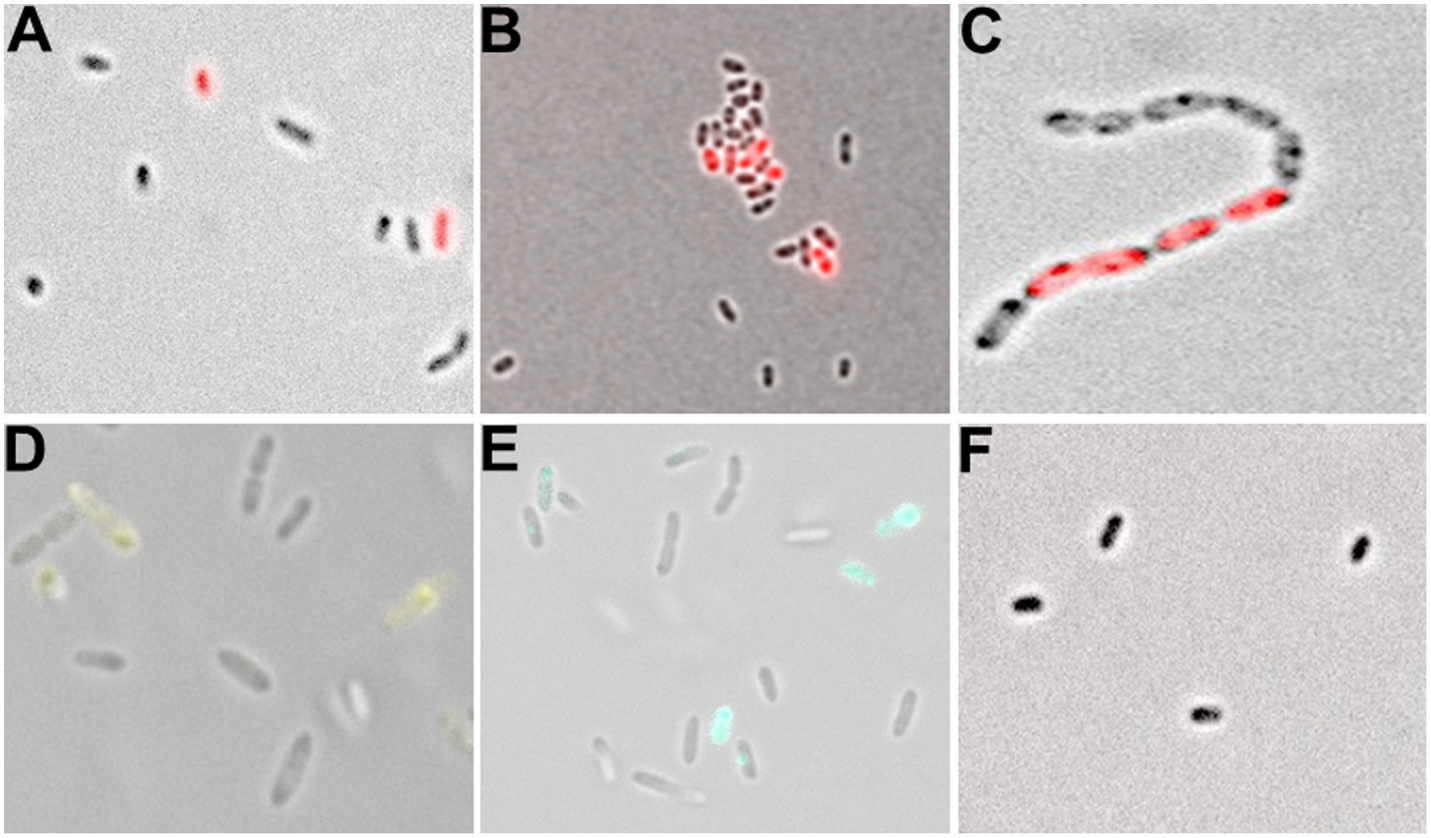
**Analysis of single cell fluorescence of P*bla*_L1_ and P*bla*_L2_ promoter gene fusions.** **(A)** Expression of the *bla*_L1_ promoter fused to *rfp* in SMK279a. Cells were grown at 30°C for 17 h under aerobic conditions (200 rpm) in LB medium containing 100 μg/ml ampicillin. Thereby, 2.0 ± 0.72% cells were in the *bla*-ON and 98.0 ± 0.72% cells were in the *bla*-OFF mode. **(B)** Expression of the *bla*_L2_ promoter fused to *rfp* in SMK279a grown under the same conditions as indicated in **(A)**. Here, 4.4 ± 0.69% of cells were in the *bla*-ON and 95.6 ± 0.69% cells were in the *bla*-OFF mode**. (C)** Phenotypic heterogeneity observed in cells (carrying the P*bla*_L2_::*rfp* fusion) forming long cell chains. Cells were grown overnight under aerobic conditions (120 rpm) in LB medium supplemented with 100 μg/ml ampicillin. **(D)** Phenotypic heterogeneity observed in cells carrying the P*bla*_L2_::*yfp* **(E)** and the P*bla*_L2_::*cfp* promoter gene fusion. **(F)** Control cells of SMK279a were grown under the same condition as described in (A) carrying a promoterless *rfp* reporter fusion (P_less_::*rfp*). Images were recorded with 63x/1.30 Oil M27 and 100x/1.30 Oil M27 lenses using a Zeiss Axio Imager 2 fluorescence microscope (Zeiss, Jena, Germany) and employing appropriate filters.

A detailed analysis of single cells by fluorescence microscopy indicated that *bla*_L1_ and *bla*_L2_ were heterogeneously expressed between individual bacterial cells (**Figure 4**). In an isogenic culture, we usually detected two stable subpopulations, i.e., individual cells in the *bla-*ON showing strong fluorescence and those in the *bla*-OFF mode that display no fluorescence. Noteworthy, individual cells within filaments (or aggregates) of exponentially growing cultures displayed a *bla*-ON mode, while adjacent cells were in a *bla*-OFF mode (**Figure 4C**). This phenomenon was observed in both cells expressing the *bla*_L1_ and the *bla*_L2_ gene fusion. Further tests in which the promoter of *bla*_L2_ was fused to a yellow and cyan fluorescent protein also confirmed heterogeneous expression of the β-lactamase within actively growing cells in the exponential phase (**Figures 4D,E**; **Supplementary Figure S3**).

Additionally, we used SMK279a carrying the native promoter of the *ampR-bla*_L2_ intergenic region fused to *rfp* (P*bla*_L2_s::*rfp*; **Table 1**). However, cells were in a *bla*_L2_-OFF mode indicating the lack of either the promoter recognition or the regulatory region that directs the expression of the reporter protein.

### RNA-seq Indicates Few Significantly Differentially Regulated Genes in Homogenous Versus Heterogeneous *bla*_L2_-Expressing Cells

**FIGURE 5:**
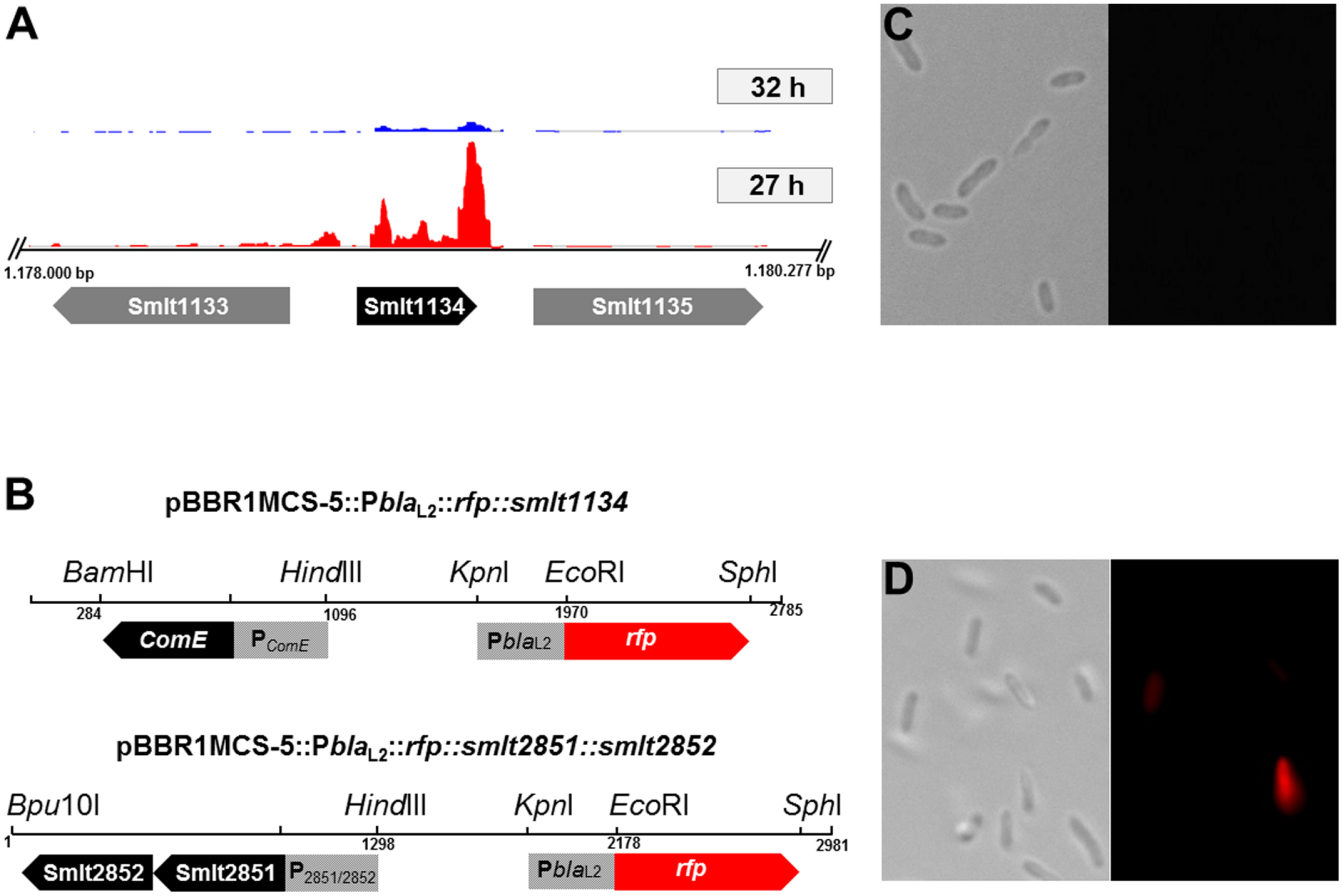
**Expression of the c*omE* homolog (*smlt1134*) under its native promoter.** **(A)** Transcriptome profiles of *smlt1134* (black arrow) and flanking genes *smlt1133*; *smlt1135* (gray arrows) in an ‘ON’ state, 32 h (blue) and an ‘OFF’ state, 27 h (red). The *comE* homolog was found to be 5.4-fold down-regulated in the ‘ON’ state (32 h). Transcriptome profile images of the leading strand and the lagging strand [generated with the IGB software (Nicol et al., 2009)] were merged and rearranged on the leading strand for a simplified visualization. **(B)** Physical map and orientation of the *comE* homolog and both putative transporter genes in pBBR1MCS-5. The promoter regions were inserted upstream of P*bla*_L2_::*rfp*, resulting in pBBR1MCS-5::P*bla*_L2_::*rfp::smlt1134*; and pBBR1MCS-5::P*bla*_L2_::*rfp::smlt2851::smlt2852.* The recombinant plasmids were transferred to SMK279a cells and challenged with 100 μg/ml ampicillin. **(C)** Expression of the *comE* homolog in SMK279a under its native promoter P*_ComE_*. Cells were cultivated for 7 h in LB medium supplemented with 100 μg/ml ampicillin. Expression of *comE* clearly affects *bla*_L2_ heterogeneous expression resulting in non-fluorescing cells (*bla*_L2_-OFF mode), here less than 1% of cells were in the *bla*-ON mode. Right and left panels are a bright-field and a fluorescence microscopic image, respectively. **(D)** Expression of both putative transporter genes (*smlt2851*; *smlt2852*) in SMK279a under their native promoter P_2851/2852_. Expression of the transporter genes did not alter phenotypic heterogeneous expression of the *bla*_L2_ gene. Here, 5.5 ± 2.12% of the cells were in the *bla*-ON and 94.5 ± 2.12% cells were in the *bla*-OFF mode. Cells were cultured under the same condition as indicated in **(C)**. Right and left panels are a bright-field and a fluorescence microscopic image, respectively. Images **(C)** and **(D)** were recorded as described in **Figure 4**.

Detailed statistical analyses of several hundred cells for each time point during growth in LB medium revealed that the majority (>90%) of the cells were in the *bla*_L2_-OFF mode during the first 24 h of growth in 25 ml batch cultures on a shaker (200 rpm). After 32 h, however, the majority of cells expressed the red fluorescent reporter and, thus, was in the *bla*_L2_-ON mode (**Table 7**). This response was independent of the presence of ampicillin. These observations prompted us to analyze the transcriptomes of cells that grew for 27 or 32 h in liquid cultures using RNA-seq. These two time points were selected because growth experiments and single cell counting indicated that less than 5% of all cells were in a *bla*_L2_-ON mode after 27 h, whereas more than 90% of all cells were in a *bla*_L2_-ON mode after 32 h. Data were analyzed as described above, and the transcriptomic metadata are summarized in **Table 5**. Two biological replicates were analyzed for each time point. Interestingly, only three genes were differentially expressed between the two time points of 27 and 32 h: *smlt2851* and *smlt2852*, encoding a putative antibiotic resistance transporter and forming an operon, and a putative *comE* homolog (*smlt1134*). The *smlt2851* and *smlt2852* genes were 29- and 7.2-fold up-regulated, respectively, while *smlt1134* was 5.4-fold down-regulated at the 32-h sampling time point (**Figure 5A**). Additional RT-qPCR data supported the observation made for the *comE* homolog. RT-qPCR indicated a 1.74 ± 0.29-fold down-regulation in the transcription of the *smlt1134* gene among cells that were in the *bla*_L2_-ON mode vs. *bla*_L2_-OFF cells. For the *smlt2851* and *smlt2852* loci, RT-qPCR data did not confirm the RNA-seq data.

**Table 7:** Effects of SMK279a P*bla*_L2_::*rfp* expression at a single cell level.

Assayed SMK279a strain	Gene copy added	^∗^% of cells showing the *bla*_L2_-ON mode after 32 h of growth
SMK279a EM2	–	>90%
SMK279a EM8	*comE* homolog	<1%
SMK279a EM9	*smlt2851-smlt2852*	>90%
SMK279aΔ*smlt1134*	–	>90%
SMK279aΔ*smlt2851*Δ*smlt2852*	–	>90%
SMK279aΔ*smlt0387*	–	>90%


*^∗^Data are mean values of three independent experiments and for each experiment we analyzed a minimum of 400 individual cells.*

To analyze the impact of Smlt2851, Smlt2852, and Smlt1134 (ComE homolog) on phenotypic heterogeneous *bla*_L2_ expression, the genes were overexpressed in SMK279a. The respective operons and predicted promoter regions were PCR amplified and inserted upstream of P*bla*_L2_::*rfp* in pBBR1MCS-5, resulting in pBBR1MCS-5::P*bla*_L2_::*rfp::smlt1134*; and pBBR1MCS-5::P*bla*_L2_::*rfp::smlt2851::smlt2852* (**Figure 5B**). The recombinant plasmids were transferred to SMK279a cells and challenged with 100 μg/ml ampicillin. Only the overexpression of the *comE* homolog in SMK279a resulted in an altered transcriptomic profile with respect to phenotypic heterogeneity at a single cell level (**Figure 5C**; **Table 7**). The expression of extra copies of the *comE* homolog in SMK279a reduced the frequency of *bla*_L2_-ON cells to 1 % or lower after 32 h of growth. The overexpression of both putative transporter genes (*smlt2851*; *smlt2852*) under their native promoter did not alter phenotypic heterogeneous expression of the *bla*_L2_ gene (**Figure 5D**). In corresponding control sets, usually >90% of all cells were in a *bla*_L2_-ON mode (**Table 7**).

Furthermore, we constructed deletion mutants of both loci, *smlt1134* (*comE* homolog) and *smlt2851*–*smlt2852* (**Supplementary Figure S4**). However, phenotypic heterogeneous gene expression of the *bla*_L2_ reporter fusion was not altered in the background of any of these deletion mutants, suggesting that the lack of expression is not linked to heterogeneous expression. Furthermore, the use of a deletion mutant strain with a mutation in the *smlt0387* gene (*ax21* homolog) did not show phenotypic heterogeneity (**Table 7**). The Ax21 peptide was suggested to act as a cell–cell signal to regulate a diverse range of functions, including virulence, in *Stenotrophomonas* (McCarthy et al., 2011). Taken together, the data indicate that the ComE homolog appears to be involved in the regulation of phenotypic heterogeneity of *bla*_L2_ gene expression in *S. maltophilia*.

## Disussion

*Stenotrophomonas maltophilia* encodes in its genome for two β-lactamases, L1 and L2. Expression of both β-lactamases is mainly controlled through the function of the master regulator AmpR in the presence of β-lactam antibiotics. The activity of AmpR is modulated via its activator (anhydro-MurNAc-oligopeptide) and repressor (UDP-MurNAc-oligopeptide) ligands (Dietz et al., 1997; Jacobs et al., 1997; Balcewich et al., 2010). Here we provide the first evidence, that beside these well-known regulatory elements, phenotypic heterogeneity plays a crucial role in β-lactamase gene expression and has profound effects on colony morphotypes. Non-genetically determined phenotypic heterogeneity has been observed in many bacteria as extensively reviewed (Ackermann, 2015; Grote et al., 2015). Well-studied systems with respect to phenotypic heterogeneity include the phenomenon of persister cells, activation of autoinducer synthase genes, flagella biosynthesis, and others (Anetzberger et al., 2009; Deris et al., 2013; Mulder and Coombes, 2013; Grote et al., 2014; Wang et al., 2014). The term ‘phenotypic heterogeneity’ thereby describes the more general phenomenon of heterogeneous behavior within isogenic cultures and is often linked with the term ‘bet-hedging’, which describes the evolutionary strategy for phenotypic heterogeneity.

The data presented in this study imply that SMK279a forms phenotypic heterogeneous subpopulations with respect to the expression of both β-lactamase genes. Two lines of evidence support this hypothesis. Firstly, cells from small and big colony variants differ mainly in the expression of 12 genes, with both β-lactamase genes *bla*_L1_ and the *bla*_L2_ being among the strongest differentially regulated genes between the two different colony morphotypes (**Table 6**). Several distinct expression patterns were stable enough to allow differentiation into different colony morphotypes. SEM analyses further revealed that many cells rather grew as long cell chains and produced OMVs in response to β-lactam treatment (**Figures 2B,C**). This phenomenon, however, was only observed in part of the populations. A second line of evidence resulted from data obtained with promoter fusions of the *bla*_L1_ and *bla*_L2_ genes. High levels of heterogeneous β-lactamase expression were observed in liquid cultures. Although, the overall number of cells that were in an ON mode varied between the different promoter fusions, they all shared the common feature of heterogeneous *bla*_L1_ and *bla*_L2_ expression (**Figures 4A–E**). Indeed, a weak and constitutive expression of the *bla*_L1_ and *bla*_L2_ genes was present in untreated cells that were grown in an environment free of stressful antibiotic challenges.

Furthermore, genome-wide sequence analysis of 24 colony variants identified a significant number of SNPs. The majority of SNPs were affiliated with genes encoding the two-component regulatory system-sensor histidine kinase Smlt3944 and the hypothetical protein Smlt1844B (**Supplementary Tables S2** and **S3**). Remarkably, none of the SNPs were observed in any of hitherto known genes that are part of the SMK279a resistome. These data imply that the colony morphotypes were indeed a result of heterogeneous behavior within a syngeneic bacterial population mainly involving genetic switches during alternations from one state to another. Furthermore, this phenotypic diversity is reversible but not based on β-lactam induced mutagenesis, since mutagenesis in bacteria has been shown to be only caused by sub-inhibitory concentration of β-lactams (Gutierrez Rodero et al., 1996). Our study also revealed that the phenotypic heterogeneity described inhere differs from previously described small colony variants (SCVs) isolated for many pathogens, including *S. maltophilia* (Proctor et al., 2006; Anderson et al., 2007). SCVs represent a subpuplation of slow-growing bacteria with growth characteristics and morphotypes distinct from those of the wild-type counterparts. Our data further indicate that ComE is part of a network involved in the regulation of phenotypic heterogeneity in SMK279a. Consequently, overexpression of the *comE* homolog resulted in a decreased heterogeneity of *bla*_L2_ expression. The *comE* gene codes for a DNA-binding protein that has been identified previously as part of a regulatory cascade responsible for bacterial heterogeneity in the Gram-positive bacterium *Streptococcus pneumoniae* (Johnsborg and Havarstein, 2009). It is noteworthy that ComE contains a helix-hairpin-helix (HhH) motif, which is mainly involved in non-sequence-specific DNA-binding (Shao and Grishin, 2000). Thereby, post-transcriptional regulation of the *comE* gene involves in part the RNA-binding protein (Hfq) that regulates a wide variety of cellular responses via interacting with small RNAs (sRNAs) and mRNAs (Roscetto et al., 2012), indicating the complex network for *comE* regulation and its diverse roles in cells. Within this framework, *comE* might also be involved in regulating phenotypic heterogeneity with respect to the *bla*_L2_ gene expression (**Figure 5C**, **Table 7**). Finally, it is important to consider that the underlying mechanism for modulating phenotypic heterogeneity is complex, involving in some cases several genes from the regulatory circuit (Morand and Mühlemann, 2007).

In summary, phenotypic heterogeneity affects β-lactamase expression in SMK279a. We speculate that phenotypic heterogeneity in SMK279a cells provides a selective advantage in natural environments and during the infection of human epithelia to respond against antimicrobial effectors. This adaptation is probably also relevant during acute and chronic human infections associated with *S. maltophilia* and effectiveness of antibiotic treatment. Future work will identify the underlying molecular switches involved in triggering phenotypic heterogeneity in *S. maltophilia*.

## Author Contributions

EA is the contributing author. EA, DK and WRS wrote and edited the manuscript. UM provided mutants and edited the manuscript. TK and SN provided the SNP analyses; KF provided the RNA-seq data; IK-M, CS, US also contributed to this research and manuscript.

## Conflict of Interest Statement

The authors declare that the research was conducted in the absence of any commercial or financial relationships that could be construed as a potential conflict of interest.

## Footnotes

1 http://hannonlab.cshl.edu/fastx_toolkit/

2 http://www.ebi.ac.uk/ena
