# Supplementary material for: Phenotypic Heterogeneity Affects Stenotrophomonas maltophilia K279a Colony Morphotypes and β-Lactamase Expression
Source: Front Microbiol. 2015 Dec 2;6:1373. doi: 10.3389/fmicb.2015.01373 (PMC4667094; doi:10.3389/fmicb.2015.01373)
Supplement: Supplementary file 1 [file Data_Sheet_1.DOCX]

***Supplementary Material***

**Phenotypic heterogeneity affects *Stenotrophomonas maltophilia* K279a colony morphotypes and β-lactamase expression**

Ebrahim M. Abda^1^, Dagmar Krysciak^1^, Ines Krohn-Molt^1^, Uwe Mamat^2^, Christel Vollstedt^1^, Konrad U. Förster^3^, Ulrich E. Schaible^2^, Thomas A. Kohl^2^, Stefan Nieman^2,4^ and Wolfgang R. Streit^1^*

*** Correspondence:**

Prof. Dr. Wolfgang R. Streit

[wolfgang.streit@uni-hamburg.de](mailto:wolfgang.streit@uni-hamburg.de)

1. **Supplementary Data**
2. **Supplementary Figures and Tables**
   1. **Supplementary Figures**

**
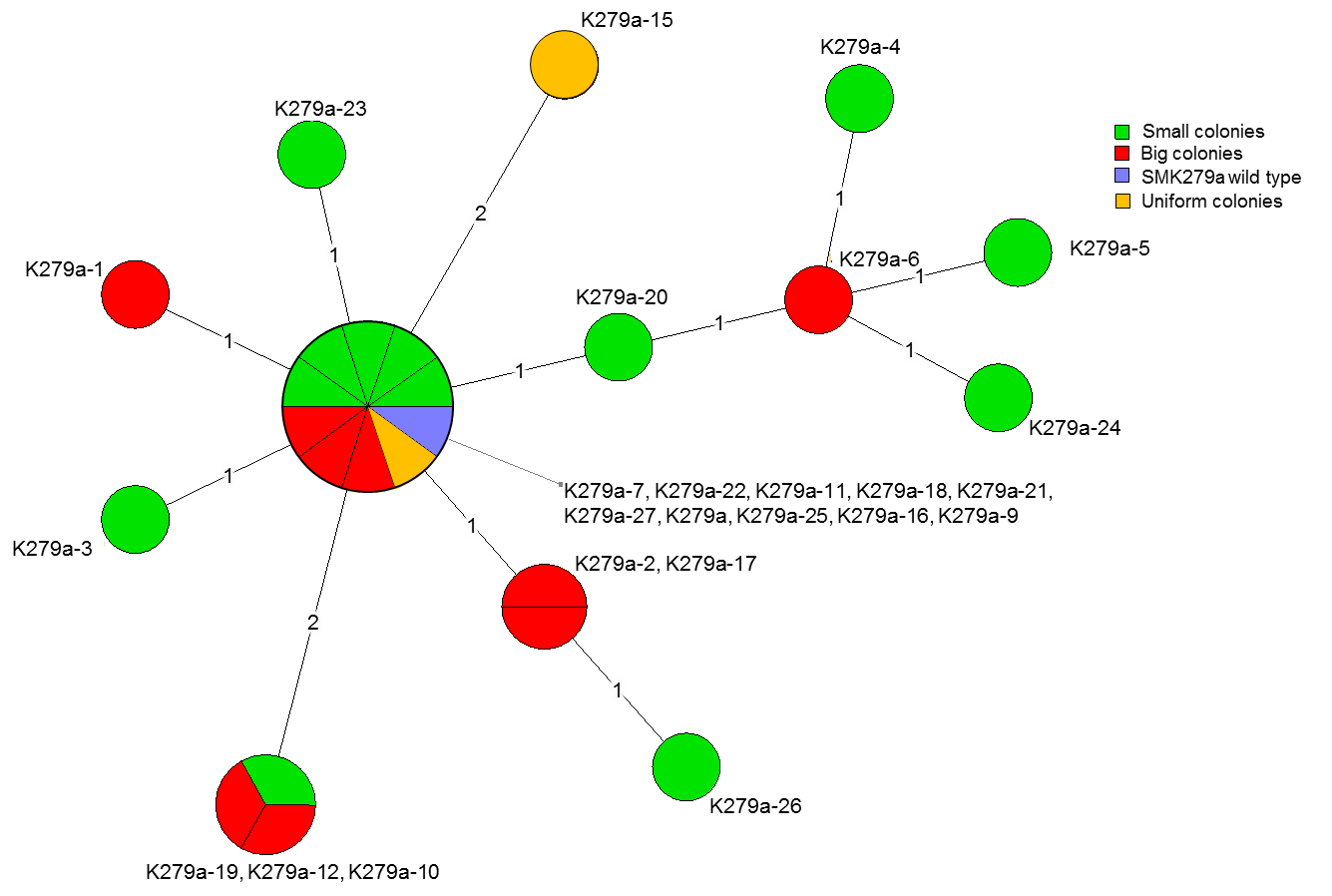
**

**Figure S1. Maximum parsimony tree built from 15 single nucleotide polymorphism positions identified in different colony variants compared to SMK279a.** The SNPs were identified in a set of 13 small, nine big and two uniform colony variants compared to the genome sequence of strain SMK279a. Numbers on branches indicate the number of distinct SNP positions between isolates. The tree was calculated with the program suite BioNumerics version 7.5 (AppliedMaths, Sint-Martens-Latem, Belgium).


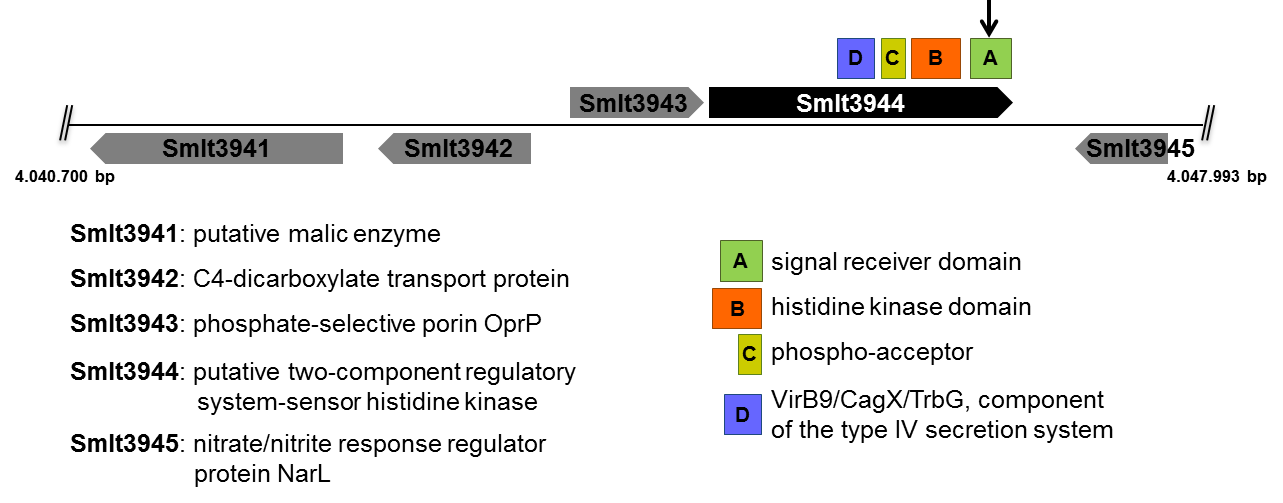


**Figure S2. Partial physical arrangement of the two component regulatory system-sensor histidine kinase Smlt3944 and flanking ORFs on the SMK279a genome.** The black arrow indicates the histidine kinase Smlt3944, grey-shaded arrows indicate flanking ORFs that are possibly under the regulatory effect of this sensor histidine kinase. Colored boxes (A-D) indicate the domains found within the sequence of Smlt3944. All SNPs identified in *smlt3944* are located within the signal receiver domain (A) of the histidine kinase and are indicated by a vertical arrow.


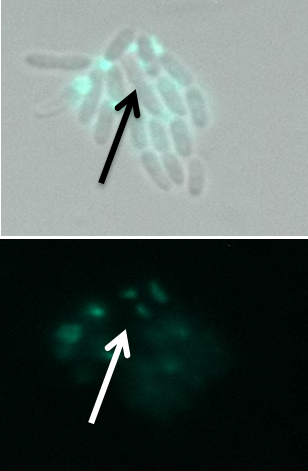


**Figure S3. Phenotypic heterogeneity at the single cell level.** Analysis of single cell fluorescence of the P*bla*_L2_::*cfp* gene fusion. Cells were grown for 2 h in the presence of gentamycin (50 µg/ml) and challenged with 100 µg/ml ampicillin. The arrows show cells in a *bla*_L2_-OFF mode. The top panel is an overlay of bright-field and fluorescence microscopic image, the bottom panel is a fluorescence microscopic image.

**
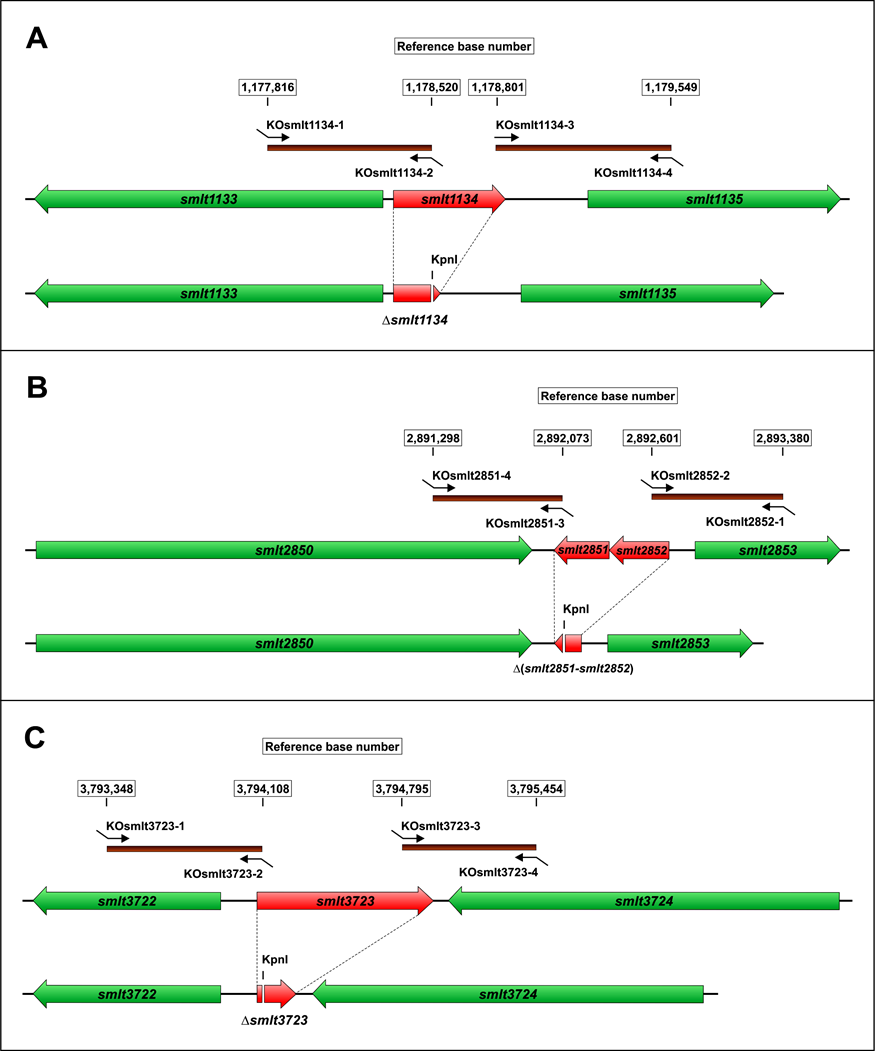
**

**Figure S4. Physical map of genes mutated in SMK279a.** Schematic representation of the cloning procedure and location of PCR fragments used to generate markerless *S. maltophilia* K279a mutants of *smlt1134* **(A)**, *smlt2851* & *smlt2852* **(B)** and *smlt3723* **(C)**. Red arrows indicate the target ORFs and their mutated versions; green arrows show the flanking ORFs; primers (Table 2) used for the amplification of PCR fragments (brown bars) are shown as thin, black arrows with labels; the necessary reference base pair numbers are framed.

**2.2 Supplementary Tables**

**Table S1.** Summary of mutations identified in SMK279a samples by whole-genome sequencing.

| **Isolate ID** | **Colony**  **morphology** | **Ampicillin [μg/ml]** | **Coverage after analysis [%]** | **SNPs** | **DEL*** | **INS*** |
| --- | --- | --- | --- | --- | --- | --- |
| K279a-1 | Big | 600 | 98.4 | 1 | 0 | 0 |
| K279a-2 | Big | 600 | 104.3 | 1 | 1 | 0 |
| K279a-3 | Small | 100 | 52.8 | 1 | 0 | 0 |
| K279a-4 | Small | 100 | 56.9 | 3 | 0 | 0 |
| K279a-5 | Small | 100 | 56.4 | 3 | 0 | 0 |
| K279a-6 | Big | 100 | 51.4 | 2 | 0 | 0 |
| K279a-7 | Big | 100 | 62.0 | 1 | 0 | 0 |
| K279a-9 | Big | 100 | 71.4 | 0 | 0 | 0 |
| K279a-10 | Big | 100 | 77.5 | 2 | 0 | 0 |
| K279a-11 | Small | 100 | 75.4 | 0 | 6 | 0 |
| K279a-12 | Small | 100 | 73.9 | 2 | 0 | 0 |
| K279a-15 | Uniform | 0 | 109.7 | 2 | 0 | 0 |
| K279a-16 | Uniform | 0 | 81.6 | 0 | 1 | 0 |
| K279a-17 | Big | 100 | 120.2 | 1 | 0 | 0 |
| K279a-18 | Big | 100 | 83.6 | 0 | 3 | 0 |
| K279a-19 | Big | 100 | 60.2 | 2 | 0 | 0 |
| K279a-20 | Small | 100 | 60.2 | 1 | 0 | 0 |
| K279a-21 | Small | 100 | 58.4 | 0 | 0 | 0 |
| K279a-22 | Small | 100 | 68.9 | 0 | 0 | 0 |
| K279a-23 | Small | 100 | 62.4 | 1 | 0 | 0 |
| K279a-24 | Small | 100 | 66.6 | 3 | 0 | 0 |
| K279a-25 | Small | 100 | 87.4 | 1 | 0 | 0 |
| K279a-26 | Small | 100 | 95.3 | 2 | 0 | 0 |
| K279a-27 | Small | 100 | 112.3 | 0 | 0 | 0 |

*DEL, deletion; INS, insertion.

**Table S2.** SNPs observed in different colony morphotypes of SMK279a. Mutations were determined comparing all 24 sequenced/analyzed colonies to our laboratory reference SMK279a genome sequence.

| **ORF/gene** | **Sum of all SNPs in colony samples*** | | | **Number of positions**  **affected within ORF** |
| --- | --- | --- | --- | --- |
|  | **small** | **uniform** | **big** |  |
| Smlt0301 | 0 | 1 | 0 | 1 |
| Smlt0590 | 0 | 0 | 1 | 1 |
| Smlt0967 | 1 | 0 | 2 | 1 |
| Smlt1450 | 1 | 0 | 0 | 1 |
| Smlt1562 | 6 | 0 | 0 | 6 |
| Smlt1844B | 27 | 3 | 35 | 7 |
| Smlt1958 | 1 | 0 | 2 | 1 |
| Smlt2315 (*flgD*) | 0 | 0 | 3 | 3 |
| Smlt2360 | 1 | 0 | 0 | 1 |
| Smlt3752 (*sucD*) | 0 | 0 | 1 | 1 |
| Smlt3885 (*mpl*) | 1 | 2 | 9 | 9 |
| Smlt3927 (*pcm*) | 1 | 0 | 0 | 1 |
| Smlt3944 | 22 | 2 | 15 | 9 |
| Smlt4235 | 4 | 0 | 1 | 1 |
| Smlt4306a2 (*cap*) | 3 | 0 | 1 | 1 |

*****Genomes of 13 small, nine big, and two uniform SMK279a colonies were analyzed.

**Table S3.** Summary of genome sequence data of *S. maltophilia* strains K279a, SMK279a∆*smlt1134*, SMK279a∆*smlt2851*∆*smlt2852* and SMK279a∆*smlt3723.*

| **SMK279a reference  base position** | **ref.**  **base** | **Strain** | | | | | | | | | | | | **Gene** | **Function** |
| --- | --- | --- | --- | --- | --- | --- | --- | --- | --- | --- | --- | --- | --- | --- | --- |
|  |  | **SMK279a wild-type control** | | | **SMK279a∆*smlt1134*** | | | **SMK279a ∆*smlt2851*∆*smlt2852*** | | | **SMK279a∆*smlt3723*** | | |  |  |
|  |  | **Type**^b^ | **Base** | **Predicted effect** | **Type**^b^ | **Base** | **Predicted effect** | **Type**^b^ | **Base** | **Predicted  effect** | **Type**^b^ | **Base** | **Predicted effect** |  |  |
| 60299 | A | DEL | Gap | Unknown | DEL | Gap | Unknown | DEL | Gap | Unknown | DEL | Gap | Unknown | None | No feature annotated; intergenic  region between *smlt0054* & *smlt0056* |
| 60300 | T | DEL | Gap | Unknown | DEL | Gap | Unknown | DEL | Gap | Unknown | DEL | Gap | Unknown | None | No feature annotated; intergenic  region between *smlt0054* & *smlt0056* |
| 70880 | T | SNP | C | Unknown | SNP | C | Unknown | SNP | C | Unknown | SNP | C | Unknown | None | No feature annotated; intergenic  region between *smlt0069* & *smlt0073* |
| 70883 | T | SNP | G | Unknown | SNP | G | Unknown | SNP | G | Unknown | SNP | G | Unknown | None | No feature annotated; intergenic  region between *smlt0069* & *smlt0073* |
| 70892 | T | DEL | Gap | Unknown | DEL | Gap | Unknown | DEL | Gap | Unknown | DEL | Gap | Unknown | None | No feature annotated; intergenic  region between *smlt0069* & *smlt0073* |
| 70893 | C | SNP | A | Unknown | SNP | A | Unknown | SNP | A | Unknown | SNP | A | Unknown | None | No feature annotated; intergenic  region between *smlt0069* & *smlt0073* |
| 502982 | T | SNP | C | Unknown | SNP | C | Unknown | SNP | C | Unknown | SNP | C | Unknown | None | No feature annotated; intergenic  region between *smlt0490* & *smlt0493* |
| 502987 | C | SNP | T | Unknown | SNP | T | Unknown | SNP | T | Unknown | SNP | T | Unknown | None | No feature annotated; intergenic  region between *smlt0490* & *smlt0493* |
| 504209 | C | SNP | A | Unknown | SNP | A | Unknown | SNP | A | Unknown | SNP | A | Unknown | None | No feature annotated; intergenic  region between *smlt0494* & *smlt0496* |
| 504211i1 | A | INS | T | Unknown | INS | T | Unknown | INS | T | Unknown | INS | T | Unknown | None | No feature annotated; intergenic  region between *smlt0494* & *smlt0496* |
| 504212 | T | SNP | G | Unknown | SNP | G | Unknown | SNP | G | Unknown | SNP | G | Unknown | None | No feature annotated; intergenic  region between *smlt0494* & *smlt0496* |
| 504215i1 | A | INS | G | Unknown | INS | G | Unknown | INS | G | Unknown | INS | G | Unknown | None | No feature annotated; intergenic  region between *smlt0494* & *smlt0496* |
| 600456 | G | DEL | Gap | Frameshift | DEL | Gap | Frameshift | DEL | Gap | Frameshift | DEL | Gap | Frameshift | *smlt0590* | GntR family transcriptional  regulator (Smlt0590) |
| 1139035 | G | SNP | A | R239H (cgc/cAc) | SNP | A | R239H (cgc/cAc) | SNP | A | R239H (cgc/cAc) | SNP | A | R239H (cgc/cAc) | *smlt1089* | Twitching mobility protein (PilT) |
| 1174560 | C | None | C | None | None | C | None | SNP | A | Unknown | SNP | A | Unknown | None | No feature annotated; intergenic  region between *smlt1129* & *smlt1130* |
| 1897615i1 | C | INS | G | Unknown | INS | G | Unknown | INS | G | Unknown | INS | G | Unknown | None | No feature annotated; intergenic  region between *smlt1846* &  s*mlt1846A* |
| 1897616 | A | SNP | T | Unknown | SNP | T | Unknown | SNP | T | Unknown | SNP | T | Unknown | None | No feature annotated; intergenic  region between *smlt1846* &  *smlt1846A* |
| 3410155 | C | None | C | None | None | C | None | SNP | T | None (tga/tAa) | None | C | None | *smlt3373* | Phenylalanyl-tRNA synthetase  subunit beta (PheT) |
| 4046251 | C | SNP | G | A840G (gcc/gGc) | SNP | G | A840G (gcc/gGc) | SNP | G | A840G (gcc/gGc) | SNP | G | A840G (gcc/gGc) | *smlt3944* | Two-component regulatory system,  sensor histidine kinase (Smlt3944) |
| 4046259i1 | G | INS | G | Frameshift | INS | G | Frameshift | INS | G | Frameshift | INS | G | Frameshift | *smlt3944* | Two-component regulatory system,  sensor histidine kinase (Smlt3944) |
| 4046260 | A | SNP | C | D843A (gat/gCt) | SNP | C | D843A (gat/gCt) | SNP | C | D843A (gat/gCt) | SNP | C | D843A (gat/gCt) | *smlt3944* | Two-component regulatory system,  sensor histidine kinase (Smlt3944) |
| 4046263 | C | DEL | Gap | Frameshift | DEL | Gap | Frameshift | DEL | Gap | Frameshift | DEL | Gap | Frameshift | *smlt3944* | Two-component regulatory system,  sensor histidine kinase (Smlt3944) |
| 4046274i1 | G | INS | G | Frameshift | INS | G | Frameshift | INS | G | Frameshift | INS | G | Frameshift | *smlt3944* | Two-component regulatory system,  sensor histidine kinase (Smlt3944) |
| 4046275 | A | SNP | T | E848V (gag/gTg) | SNP | T | E848V (gag/gTg) | SNP | T | E848V (gag/gTg) | SNP | T | E848V (gag/gTg) | *smlt3944* | Two-component regulatory system,  sensor histidine kinase (Smlt3944) |
| 4177234 | C | None | C | None | SNP | T | V575M (gtg/Atg) | None | C | None | None | C | None | *smlt4071* | Acriflavin resistance protein B (AcrB) |
|  |  |  |  |  |  |  |  |  |  |  |  |  |  |  |  |
| ^a^Table shows all variations identified by mapping of the sequence reads to the *S. maltophilia* K279a genome sequence [GenBank: NC_010943.1]. Uncovered regions corresponding to the deleted *smlt1134*, *smlt2851* & *smlt2852* and *smlt3723* genes are not included. | | | | | | | | | | | | | | | |
